# Platelet activation determines the severity of thrombocytopenia in dengue infection

**DOI:** 10.1038/srep41697

**Published:** 2017-01-31

**Authors:** Amrita Ojha, Dipika Nandi, Harish Batra, Rashi Singhal, Gowtham K. Annarapu, Sankar Bhattacharyya, Tulika Seth, Lalit Dar, Guruprasad R. Medigeshi, Sudhanshu Vrati, Naval K. Vikram, Prasenjit Guchhait

**Affiliations:** 1 Disease Biology Laboratory, Regional Centre for Biotechnology, National Capital Region Biotech Science Cluster, Faridabad, India; 2 Department of Biotechnology, Manipal University, Karnataka, India; 3 Vaccine and Infectious Disease Research Center, Translational Health Science and Technology Institute, National Capital Region Biotech Science Cluster, Faridabad, India; 4 Department of Medicine, All India Institute of Medical Sciences, New Delhi, India; 5 Department of Microbiology, All India Institute of Medical Sciences, New Delhi, India

## Abstract

Thrombocytopenia is common in patients with dengue virus (DENV) infections. With a focus on understanding the possible mechanism of thrombocytopenia in DENV infections we described a direct correlation between activation and depletion of platelets in patients. Our data showed a sharp decrease in platelet counts at day 4 of fever in patients. The high DENV genome copies in platelets correlated directly with the elevated platelet activation along with increased binding of complement factor C3 and IgG on their surface at day 4. Recovery in platelet count was observed on day 10 through day 6 and 8 with simultaneous decrease in platelet activation markers. Further, our *in vitro* data supported the above observations describing a concentration-dependent increase in platelet activation by DENV serotype-2. The high copy number of DENV2 genome in the platelet pellet correlated directly with platelet activation, microparticle generation and clot formation. Furthermore the DENV2-activated platelets were phagocytosed in large numbers by the monocytes. The DENV2-mediated lysis and clearance of platelets were abrogated in presence of platelet activation inhibitor, prostacyclin. These observations collectively suggest that platelet activation status is an important determinant of thrombocytopenia in dengue infections. A careful strategy of inactivation of platelets may rescue them from rapid destruction during DENV infections.

Dengue virus (DENV) infects an estimated 390 million people and causes 25000 deaths each year around the world. All four serotypes of DENV (1–4) are reported to cause the disease transmission primarily in tropical and subtropical countries including India^1^,^2^,^3^. The spectrum of illness of this infection can range from a mild febrile illness to the severe forms of the disease, such as dengue hemorrhagic fever (DHF) and dengue shock syndrome (DSS). Clinically dengue infection follows three distinct phases of illness i.e febrile, critical and recovery. The initial symptoms are common to all patients, which includes high fever, body pain, headache, rashes and occasional bleeding during day 2–7 of infection, while the severe forms of the disease evolve rapidly with symptoms such as hypotension, shock, edema and vascular leakage^4^,^5^,^6^.

Thrombocytopenia is another clinical manifestation and is common in patients with either mild or severe cases of DENV infections. Studies suggest that the low platelet count is one of the major causes of bleeding in these patients. The platelet counts drop below normal level (150,000–450,000 platelets/μL) and may reach as low as <40000 platelets/μL during day 3–7 of fever in many patients^6^. In some cases, patients have to be transfused with platelets to maintain their normal hemostatic activity^7^,^8^. Development of thrombocytopenia in dengue patients mainly rests on two events: decreased production of platelets in the bone marrow and/or increased destruction and clearance of platelets from peripheral blood^9^. Several studies suggest that the activation and dysfunction of platelets is implicated in the prothrombotic complications in DHF and DSS^10^,^11^. Studies report that the platelet activation [with elevated surface P-selectin] and apoptosis [with increased caspases and phosphatidylserine (PS) expression] are associated in the early days of dengue infection^12^,^13^. Reports also show that the activation of complement factor C3 followed by binding of C5b-9 complex to platelet surface is significantly linked with platelet destruction and thrombocytopenia in these patients^14^,^15^,^16^. Further an *ex vivo* report describes a direct correlation between microparticles (MPs) derived from activated platelets in the peripheral blood and the severity of thrombocytopenia in dengue patients^17^.

The DENV can infect cells of diverse lineage including monocytes, macrophages, dendritic cells and skin Langerhan cells^18^. DENV also infects circulating platelets and their progenitor megakaryocytes in bone marrow^19^,^20^. It interacts with platelets and megakaryocytes *via* the cell surface receptor FcγRII^21^. A recent study describes that DENV can bind directly to surface receptors such as DC-SIGN and heparan sulphate proteoglycan and enter the platelet, where they replicate and propagate successfully^22^,^23^.

Although the mechanisms involved in immune mediated destruction and clearance of platelets in dengue infections have been explored, several queries remain unclear. Our study focused on investigating whether the platelet activation status is an important determinant of platelet destruction and clearance. Our data showed that circulating platelets carrying high copy numbers of DENV genome were hyperactive expressing elevated P-selectin and binding of PAC-1 (activation-dependent antibody) on surface and generating MPs (well known makers of platelet activation)^12^,^13^,^24^. These activated platelets displayed elevated levels of surface bound C3 and IgG. Further, our *in vitro* data also supported the above observations demonstrating the significant activation of platelets upon treatment with DENV but not by another flavivirus, Japanese Encephalitis virus (JEV). Furthermore we observed that the DENV-mediated activation of platelets was significantly abrogated in the presence of platelet activation inhibitor such as prostacyclin (PGI2).

## Results

### Low platelet counts coexisted with the high platelet activation and vice versa during different days of infection in dengue patients

**Figure 1:**
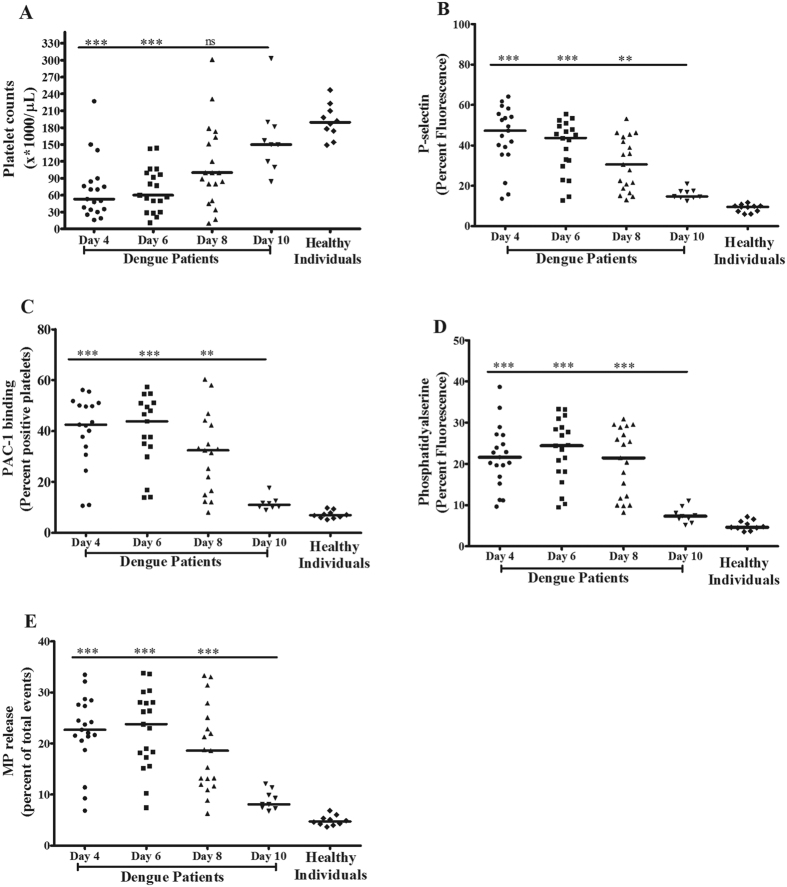
Platelet counts and platelet activation during different days of fever in dengue patients. **(A)** Platelet number was measured from freshly isolated blood samples of dengue patients (n = 19) at different days of fever (4, 6, 8 and 10) and of healthy controls (n = 10). Dot represents median value for each individual from three separate readings. Low platelet count was observed at day (d) 4 and rescued at d10, ***p < 0.0007 d4 vs d10, ***p < 0.0003 d6 vs d10, ns = non-significant d8 vs d10 (Mann-Whitney test). **(B)** P-selectin expression, **(C)** PAC1 binding **(D)** PS exposure on platelets were measured from freshly isolated platelets from patients and normal individuals by flow cytometry. All three parameters show elevated level at d4 and decreased further at d10 (P-selectin: ***p < 0.0003 d4 vs d10, ***p < 0.0004 d6 vs d10 and **p < 0.0027d8 vs d10; PAC1: ***p < 0.0005 d4 vs d10, ***p < 0.0002 d6 vs d10 and **p < 0.0018 d8 vs d10; PS: ***p < 0.0001, d4/6/8 vs d10). **(E)** Platelet-derived microparticles (MPs) in plasma were measured by flow cytometry using CD41a antibody. Plasma MPs level was highest at d4 and lowest at d10 (***p < 0.0004 dv vs d10, ***p < 0.0002 d6 vs d10 and ***p < 0.0008 d8 vs d10). Data of healthy individuals is mentioned as normal reference. The detail of each patient’s data is mentioned in suppl. Table-1.

To elucidate the association between thrombocytopenia and activation status of platelets in peripheral blood of the dengue patients, the platelet counts along with platelet surface activation markers such as P selectin and PS expression, and PAC1 binding were measured at various days of fever. Study also measured the platelet-derived microparticles (MPs) in patients’ plasma. The low platelet counts (mean < 50000/μL) was observed at day 4 of fever, which recovered to normal level at day 10 (>170000/μL), (Fig. 1A). In contrast, all four platelet activation markers showed higher values during day 4 and 6, and decreased further to lower levels at day 10 of fever (Fig. 1B–E). The correlation analysis between platelet count vs. platelet P-selectin (r = −0.807)/PAC1 (r = −0.812)/ PS (r = −0.741)/ platelet-MPs (r = −0.8075) expression during different days of fever showed the inverse association (Suppl. Fig. 1). The patients’ data are mentioned in detail in Suppl. Table-1.

### High copies of DENV genome existed in activated platelets in patients

**Figure 2:**
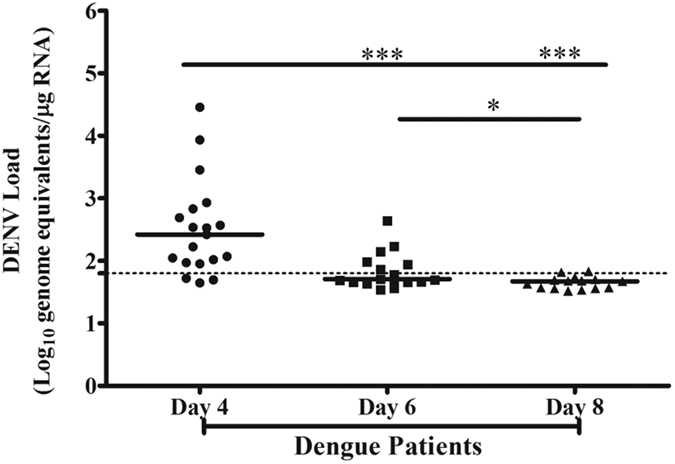
DENV genome copies in platelets during different days of infection in patients. The copy number of DENV genome was measured in platelet pellets of patients using qPCR. Data are calculated as mentioned in Fig. 1. The high copy number of DENV genome in platelets at d4 was further decreased at d6 and d8, ***P < 0.0006 d4 vs d6, ***p < 0.0001 d4 vs d8, *p < 0.0414 d6 vs. d8 (using Mann Whitney Test). Dotted line represents the limit of detection.

Furthermore data show that the presence of high copy numbers of DENV genome in platelet pellet correlated directly with the platelet activation markers during day 4, 6 and 8 (Fig. 2). The copy numbers of DENV genome correlated with platelet P-selectin expression, r = 0.601 during all above day points, and separately at day 4 (r = 0.764), day 6 (r = 0.487) and day 8 (r = 0.546); with PAC-1 binding during all days (r = 0.372), separately at day 4 (r = 0.509), day 6 (r = 0.3) and day 8 (r = 0.18); with PS expression during all days (r = 0.238), separately at day 4 (r = 0.617), day 6 (r = 0.327) and day 8 (r = 0.353); with platelet-MP level in plasma during all days (r = 0.357), separately at day 4 (r = 0.24), day 6 (r = 0.384) and day 8 (r = 0.428); (Suppl. Fig. 2).

### Increased binding of complement factor C3 and IgG to activated platelets in patients

**Figure 3:**
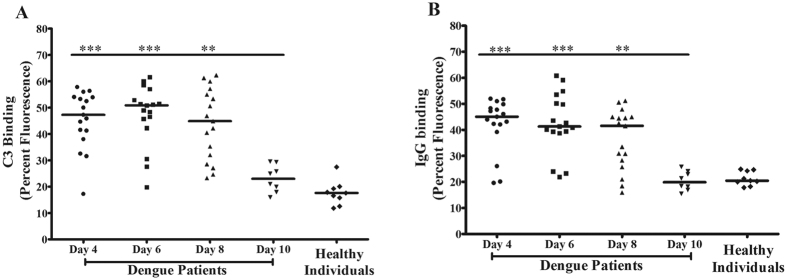
Platelet surface bound complement factor C3 and IgG in patients: **(A)** C3 and **(B)** IgG binding to platelet surface was measured by flow cytometry and calculated (using Mann Whitney Test) as mentioned in Fig. 1. Highest levels of both C3 and IgG were measured at d4 and lowest at d10 (C3: ***p < 0.0004 d4 vs d10, ***p < 0.0005 d6 vs d10, **p < 0.0012 d8 vs. d10; IgG: ***p < 0.0005 d4 vs d10, ***p < 0.0004 d6 vs d10, **p < 0.0027 d8 vs d10).

Since C3 and IgG are known to bound to the surface of activated-platelets and also known for their role in lysis and clearance of platelets, this study further measured the binding of complement factor C3 and IgG on the platelet surface in patients during different days of fever. Data show the elevated binding of both C3 and IgG to platelets when platelets existed at hyperactive conditions during day 4 and 6, which was further decreased at day 10 (Fig. 3A,B) indicating the possible mechanism of complement-antibody mediated clearance as well as lysis of the circulating platelets in patients.

### DENV2 triggered activation and apoptosis of platelets *in vitro*

**Figure 4:**
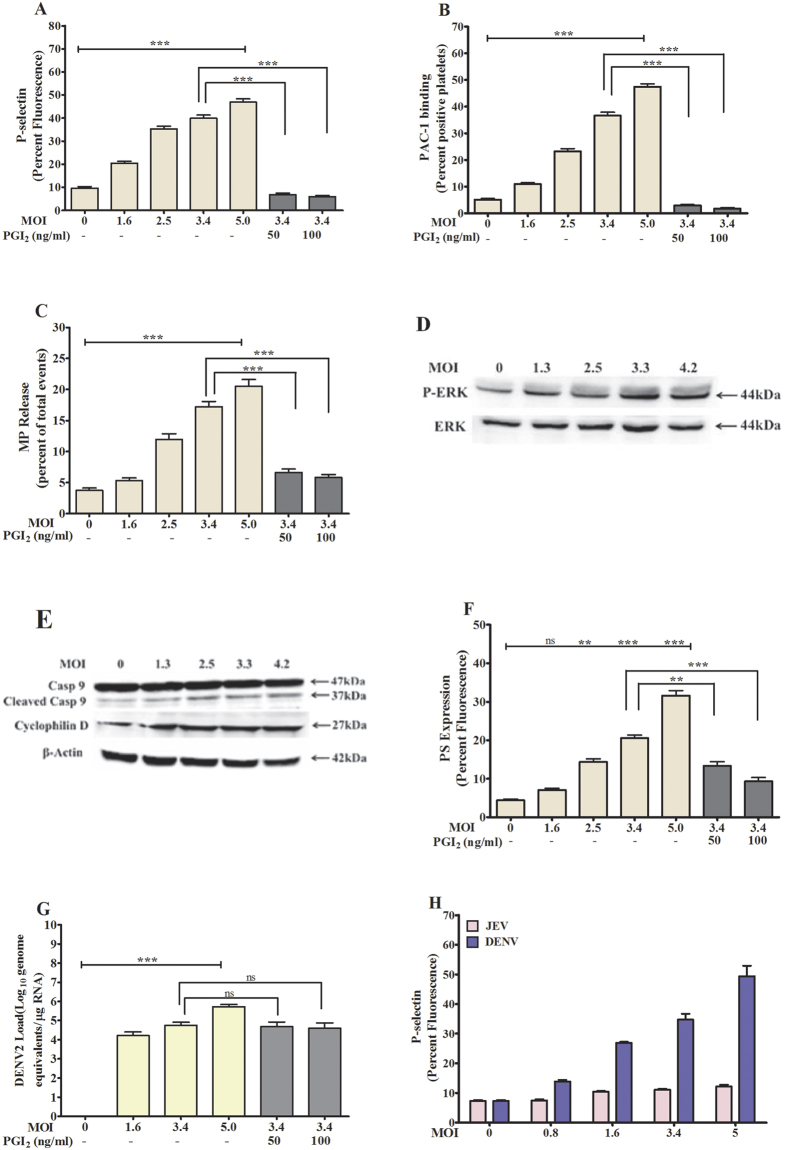
Platelet activation and apoptosis mediated by DENV2 *in vitro*. Platelet rich plasma (PRP) was incubated with different MOI of DENV2. **(A)** P-selectin expression and **(B)** PAC-1 binding on platelet, and **(C)** platelet-derived MPs from supernatant were measured by flow cytometry. Data are the mean ± SEM of percent fluorescence from three different experiments. The DENV2 treatment increased both parameters in a concentration-dependent manner, ***p < 0.0001 (one way ANOVA). The above expressions were significantly inhibited by the platelet activation inhibitor PGI2 (50 and 100 ng/mL) ***p < 0.0001 (Student’s t-test). The DENV2 treatment has increased the **(D)** phosphorylation of ERK and **(E)** activation of caspase 9 (activation generates 37 kDa cleaved band) and cyclophilin D in platelets in a concentration-dependent manner, measured by immunoblotting. The densitometry data and uncut immunoblots of pERK/ERK, caspase 9 and cyclophilin D are mentioned in Suppl. Fig. 4(A–H). **(F)** DENV2 also increased PS expression in a concentration-dependent manner, **p < 0.001 and ***p < 0.0001. Further, both concentrations of PGI2 inhibited the PS expression significantly, **p < 0.002 and ***p < 0.0003. **(G)** The qPCR data show the higher number of DENV2 genome copy in platelet pellets when PRP was treated with higher MOI of DENV2, ***p < 0.0001, NS = non-significant. **(H)** P-selectin expression on platelets was measured following the treatment of PRP with different MOI of Japanese Encephalitis Virus (JEV) and DENV2. DENV2 activated platelets in a concentration-dependent manner, p < 0.001, while JEV did not show significant effect. The data for PAC1 binding, PS expression on platelets and platelet-MPs generation by DENV and JEV are mentioned in Suppl. Fig. 5.

Study further examined the activation of platelets by the dengue virus serotype 2 (DENV2) *in vitro.* Our data show that when the platelet rich plasma (PRP) was incubated with various MOIs of DENV2, the virus triggered the platelet activation in a concentration-dependent manner elevating P-selectin expression (Fig. 4A), PAC1 binding (Fig. 4B), platelet-MPs generation (Fig. 4C) and also the phosphorylation of signaling adapter kinase such as ERK in platelets (Fig. 4D). Furthermore our data show that the platelet inhibitor, prostacyclin (PGI2) significantly inhibited the DENV2-mediated platelets activation by decreasing the expression of the above platelet activation markers (Fig. 4A–C). Our data further describe the activation of pro-apoptotic proteins such as caspase 9 and cyclophilin D (Fig. 4E), and the surface expression of PS in platelets induced by DENV2 in a concentration-dependent manner (Fig. 4F). Study also examined the specificity of dengue virus showing the concentration-dependent activation of platelets by DENV but not by another flavivirus, Japanese Encephalitis (JE), Fig. 4H, Suppl. Fig. 5.

### DENV2 treatment increased C3 and IgG binding to platelet

**Figure 5:**
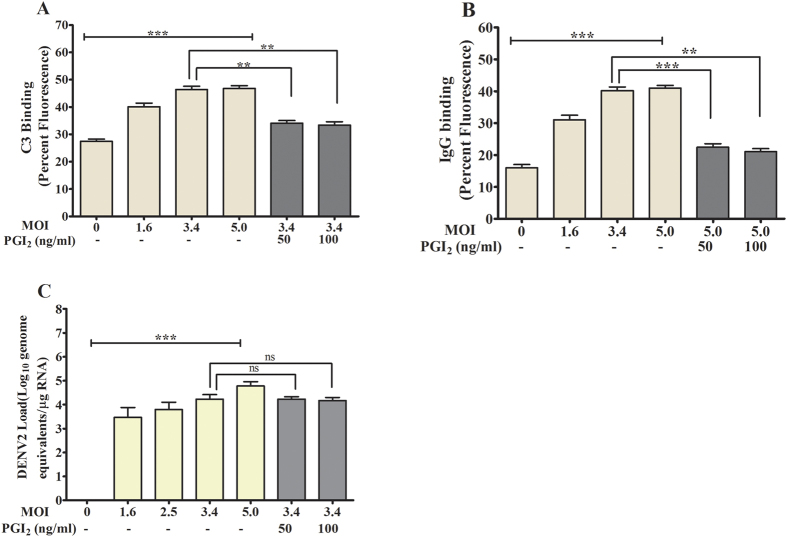
Complement factor C3 and IgG binding on platelets treated with DENV2 *in vitro*. **(A)** C3 and **(B)** IgG bound to platelet surface (measured by flow cytometry and analyzed as mentioned in (Fig. 3) a concentration-dependent manner when washed-platelets were treated with DENV2 in presence of 20 μL of plasma (plasma was used as the source of C3/IgG in total reaction volume of 400 μL), ***p < 0.0001. The above binding of C3 (**p < 0.003) and IgG (**p < 0.001, ***p < 0.0007) to platelets was abrogated in presence of PGI2. **(C)** The qPCR data show the higher number of DENV genome copy in platelet pellets when incubated with higher MOI of DENV2, ***p < 0.0001.

Study further examined the binding of complement factor and immunoglobulin on platelet. Our data showed that both complement factor C3 and IgG bound to platelet surface in an activation-dependent manner upon treatment with DENV2. Furthermore the inhibition of platelet activation by PGI2 abrogated the C3 and IgG binding on platelets (Fig. 5A,B).

### DENV2 induced platelet clot or thrombus formation *in vitro*

**Figure 6:**
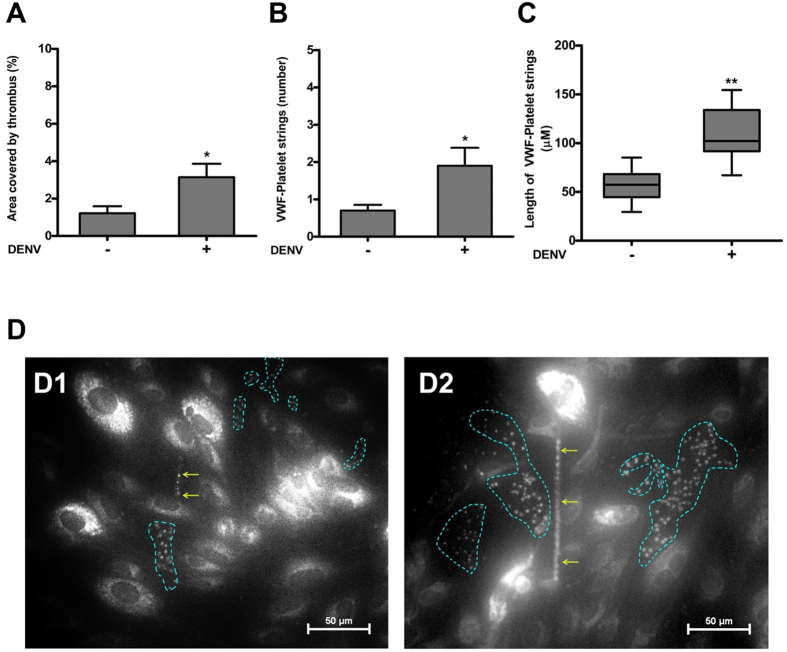
Platelet thrombus and platelet-VWF string formation on HUVEC monolayer under flow shear *in vitro* in presence of DENV2. Whole blood of healthy individual was incubated with or without DENV2 (3.4 MOI) and perfused over HUVEC monolayer under shear of 2.5 dyne/cm^2^ in presence of mepacrine (label platelets). **(A)** Platelet thrombus area and **(B)** platelet-VWF string number and **(C)** string length were measured. Data are the mean ± SEM from ten view-fields in three independent experiments. The thrombus area, and string number and length were increased in presence of DENV2, *p < 0.05. The string length (calculated as median with highest and lowest value) was increased in presence of DENV2, **p < 0.005. **(D)** The 40X images show the platelet thrombus (marked by dotted area) and platelet- VWF string (marked by arrow) in absence **(D1)** and presence **(D2)** DENV2.

Since the clot/thrombus formation is an important cause of platelet clearance and our above data have shown that the activation of platelets was mediated by DENV *in vivo* and *in vitro*, study therefore examined the prothrombotic functions of these DENV-activated platelets. Our data show that the DENV2 treatment to platelet-rich plasma significantly increased the platelet clot formation (as measured by decreased clot formation time) *in vitro* in an activation-dependent manner Suppl. Fig. 6. Furthermore the DENV2 treatment to whole blood also exhibited an elevated platelet thrombus formation on histamine-activated HUVEC monolayer under flow condition. The size of the platelet thrombus, the number and length of platelet-VWF strings on the endothelium were increased significantly following DENV2 treatment (Fig. 6). In our earlier works, we have shown that the formation of platelet thrombus^25^ or platelet-VWF strings^26^ on subendothelial matrix proteins or endothelium monolayer significantly depletes platelets from flowing blood. Therefore it is possible that the depletion of platelets from peripheral blood in dengue patients could be due to the increased adhesion of hyperactive platelets to the endothelium of the injured-blood vessels.

### DENV2-treated platelets were phagocytosed rapidly by monocytes *in vitro*

**Figure 7:**
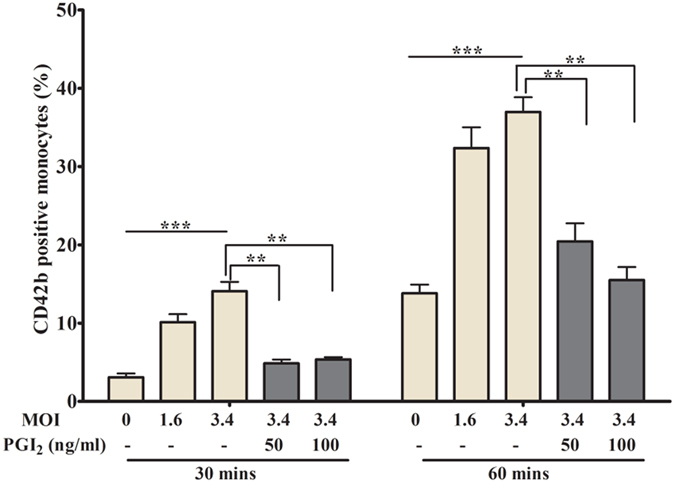
Phagocytosis of DENV2 activated platelets by monocytes: The washed-platelets treated with various MOI of DENV2, were exposed to monocytes (CD14^+^) in presence of autologous plasma. The platelet marker CD42b was measured in monocyte by intracellular staining and flow cytometry, and presented as mean ± SEM of percent fluorescence of three independent experiments. Platelets treated with increasing MOI of DENV2 were phagocytosed more by the monocytes at 30 and 60 min, ***p < 0.001. The phagocytosis of platelets by monocytes was abrogated when platelets were inactivated in presence of PGI2,**p < 0.005.

Our study also examined the clearance of activated-platelets by the phagocytic cells such as monocytes. As expected our data show that the DENV2-treated platelets were engulfed more rapidly by the monocytes. Furthermore the inhibition of platelet activation by PGI2 abrogated the platelet engulfment by monocytes (Fig. 7) suggesting that the activation of status of platelets during DENV infection determines the platelet clearance by phagocytic cells.

## Discussion

Thrombocytopenia is a common clinical manifestation of dengue infection. With a focus on investigating the various mechanisms of thrombocytopenia in dengue infection, we described from this study: 1) the activation status of platelets was a crucial cause of their depletion from circulation in patients with DENV infections and 2) the mechanism of platelet depletion included processes such as lysis, aggregation and clot formation and phagocytosis of platelets. Although other studies have reported the association between platelet activation and thrombocytopenia in dengue infection^11^,^12^,^13^, our data specifically described a very clear association between activation status of platelets and their destruction/depletion from circulation in patients over the period of fever, beginning at day 4 of fever when they were admitted to the hospital with low platelet count (<50000/μL) and followed up till the platelet count recovered to normal level by day 10 (>170000/μL). Furthermore our data showed that the circulating platelets existed in the hyperactive states with maximum expression of P-selectin and PS, PAC1 binding and an elevated level of platelet-MPs in plasma on day 4 of fever, which returned to the normal range by day 10. The activated platelets from patients had high levels of surface-bound complement factor C3 and IgG on day 4–6 of fever indicating the possibility of complement complex C5b-9 mediated lysis of the hyperactive platelets^16^ as well as clearance of the IgG-bound platelets by phagocytic cells^27^ in patients. Interestingly, the platelet pellet from patients on day 4 contained a relatively high copy number of DENV genome indicating a direct correlation between viral loads in platelets and their activation status. Several studies have described the mechanisms by which DENV binds to platelet surface receptors such as FcγRII^21^, DC-SIGN and heparan sulphate proteoglycan^22^,^23^ and enters into cells, where they replicate and propagate.

Further our *in vitro* data also supported the above *in vivo* observations. The incubation of platelet rich plasma or washed platelets with dengue virus serotype 2 (DENV2) triggered the activation and apoptosis of platelets in a concentration-dependent manner. The DENV2 genome copy number in platelet pellet correlated directly with platelet activation in a concentration-dependent manner. Our data also showed that the platelet activator inhibitor PGI2 significantly abrogated the DENV2-mediated platelet activation and apoptosis. Furthermore our data showed that DENV2-treated platelets promoted significantly the platelet clot formation in an activation-dependent manner. *In vitro*, perfusion of DENV2-infected blood over the activated-endothelium monolayer under physiological flow shear condition formed significantly large platelet thrombus as well as elevated number of platelet-VWF strings, indicating a mechanism of platelet depletion from circulation of these patients particularly when the platelets exist in hyperactive states during infections. Other studies also have reported that the increased adhesion of platelets to endothelium contributed to the depletion of platelets and thrombocytopenia in DHF patients^28^. Furthermore our data showed that the monocytes engulfed DENV2-treated platelets rapidly in an activation-dependent manner. Further, an inhibition of platelet activation significantly abrogated the platelet phagocytosis by the monocytes indicating that the activation status of platelets during DENV infection could be an important determinant for the platelet clearance by phagocytic cells. Likewise, other studies also have reported the presence of a significant number of monocytes and macrophages with ingested platelets in dengue patients^29^,^30^. Studies also have reported that the engulfment of activated-platelets by phagocytic cells such as monocytes, dendritic cells and neutrophils contributed to platelet depletion in dengue infections^31^,^32^.

Therefore our above observations together suggest that platelet activation state is an important determinant for platelet lysis and clearance and thus thrombocytopenia in dengue patients. This study also suggests that a careful strategy of inactivation of platelets may rescue them from rapid destruction and depletion during DENV infections.

## Material and Methods

Antibodies, phospho (P)-AKT (S473) and caspase 9 from the Cell Signaling (Beverly, MA, USA) and cyclophilin D (Cloud Clone Corp. USCN, Wuhan, China) were purchased. The anti β-actin antibody was purchased from the Thermo Scientific, Rockford, USA. Immunofluorescence antibodies such as anti- human CD62P FITC (R&D systems, Minneapolis), anti-human CD41a PE, CD42b APC, annexin V FITC and PAC1 FITC antibodies (BD Biosciences, San Jose, USA), anti-human IgG PE and anti-human C3 FITC (eBiosciences, San Diego, USA) were used. All methods were performed in this study in accordance with the relevant guidelines and regulations.

### Dengue Patients

To obtain blood samples, approval was obtained from the Institutional Ethics Committee for Human Research of Regional Centre for Biotechnology (RCB) (Ref. No. RCB-IEC-H-3, 31.07.2014) as well as from All India Institute of Medical Sciences (AIIMS), New Delhi (Ref. No. IEC/NP-39/13.03.2014). Informed consent was provided according to the recommendations of the declaration of Helsinki. 5 mL of blood sample was collected in ACD anticoagulant at different days of infections from enrolled patients. A total of 46 dengue patients were recruited at AIIMS. Only 19 of them were followed for 3 (day 4, 6 and 8 of fever) or 4 (day 4, 6, 8 and 10 of fever) time-points for collecting blood samples. The first sample was collected from all patients between day 3 and 4 of fever while they were admitted to the critical care unit of AIIMS with suspected dengue infections. All patients were recruited without major clinical complications other than symptomatic fever and low platelet counts. 16 of 19 patients were detected to be NS1 positive and the other 3 patients had DENV specific IgM. Other patients have either withdrawn participation from study, or sample collection was not possible due to medical condition, or other reasons. Clinical data including platelet counts were collected for all patients. All patients were strictly advised against use of Aspirin and other NSAIDs. Only Paracetamol was prescribed/administered for management of fever. Platelet surface markers such as P-selectin, PAC-1, PS, C3, IgG and platelet-derived MPs were measured from the samples. The healthy volunteers (n = 10) were recruited at RCB, 5 ml blood sample was collected at one time, and all the above parameters were measured and used as normal reference. The healthy individuals did not have history of dengue infections in recent years.

### Platelet counts

Platelet counts were measured from patients’ whole blood at the Hematology Department of AIIMS using the automated cell counter, Sysmex XT-1800i (Diamond Diagnostics, USA).

### Virus Preparation

Dengue virus serotype 2 (DENV2) strain NGC was expanded in C6/36 cells, cultured in L15 media supplemented with 10% FBS and standard concentrations of penicillin-streptomycin-glutamine^13^,^14^. Briefly, cells were infected using DENV2 inoculum diluted in L15 supplemented with 2% FBS for 2 hours at 37 °C, 5% CO_2_ with continuous gentle rocking. At the end of infection the inoculum was discarded and complete L15 media overlaid on the cells. The infected cells were incubated at 37 °C under 5% CO2 for 5 days. Subsequently the supernatant was collected and clarified by centrifugation at 1000 RPM for 10 minutes. The clarified cell supernatant was aliquoted and stored at −80 °C for storage. The amounts of infectious particles were expressed as fluorescence forming units (FFU)/mL and titrated in Vero cells. Platelets were infected with DENV2 at different multiplicity of infections (MOI) i.e. 0, 1.6, 2.5, 3.4 and 5 for the experiments. The range of MOI of DENV2 used for *in vitro* study was similar to the MOI observed in dengue patients’ blood as mentioned in Suppl. Fig. 7. The Japanese Encephalitis virus (JEV) was grown in Porcine kidney cells^33^ and the virus titre was estimated as mentioned above.

### Quantification of virus

The RNA was isolated from washed pellets of platelet (platelet counts were adjusted to approximately 300 × 10^3^/μL) collected from patients during day 4, 6 and 8 of fever using Ambion TRIzol Reagent (Thermo Fisher Scientific). The RNA was eluted in 30 μL of DEPC treated water and stored at −80 °C. Equal amount of RNA (2 μg) was reverse transcribed using the reverse primer with SuperScript IV First-Strand Synthesis System (Invitrogen, Thermo Fisher Scientific). For 10 μl final reaction volume, 1.6 μL of cDNA of each sample was used for qPCR using 200 nM of forward primer, 300 nM reverse primer, 250 nM probe and TaqMan universal PCR master mix (Applied Biosystems). The amplification was performed with pre incubation of above reaction mixture at 50 °C for 2 min, denaturation at 95 °C for 10 min followed by 45 cycles of 95 °C for 15 sec and 60 °C for 1 min using the Applied Biosystems 7500 real-time PCR system. The forward primer 5′-GARAGACCAGAGA TCCTGCTGTCT-3′ and reverse primer 5′-ACCATTCCATTTTCTGGCGTT-3′ used for detection of the viral genome^15^. The copy number of DENV genome was determined as mentioned^34^,^35^. The copy numbers of DENV below the detection level and above the half-detection level were included for statistical analysis.

### Measurement of platelet surface markers

The platelet surface P-selectin, PAC1, phosphatidyalserine (PS), C3 and IgG was measured using flow cytometry assay as mentioned in our recent work^24^. Within 2–3 hours after the collection of blood samples, the platelet rich plasma (PRP) was isolated from whole blood by centrifugation. The platelets were labeled with either anti-P selectin FITC, annexin V FITC, PAC1 FITC, anti-C3 FITC or anti-IgG PE antibodies by incubating for 30 min at RT and fixed with 1% paraformaldehyde for flow cytometry analysis (Becton Dickinson, San Jose, USA). The above values were normalized with assay control. A single stock of fixed lyophilized platelets of normal individual was run as assay control along with all patients’ samples, and data were normalized accordingly. In *vitro*, washed platelets were incubated with various concentrations of DENV2 for 45 min at 37 °C and the above platelet surface markers were measured using flow cytometry.

### Microparticle measurement

Platelet microparticles (MPs) were measured from patients’ plasma using flow cytometry. Platelet MPs of all samples were measured together. Frozen plasma were thawed at 37 °C water bath and centrifuged at 1500 g for 15 minutes and supernatant was used for measuring platelet derived MPs using anti- CD41 PE antibody. To measure MPs we followed a uniform gating strategy as mentioned in our recent work^24^. *In vitro*, washed platelets were incubated with different concentrations of DENV2 and supernatant was collected for MP measurement using flow cytometry as mentioned above.

### Washed platelet preparation

Platelet-rich plasma (PRP) was isolated from blood collected from normal healthy donors in acid-citrate dextrose (ACD) anticoagulant. Further, PRP was centrifuged, and the platelets were resuspended in calcium-free Tyrode buffer [126 mM NaCl, 2.7 mM KCl, 1 mM MgCl_2_, 0.38 mM NaH_2_PO_4_, 5.6 mM dextrose, 6.2 mM sodium HEPES, 8.8 mM HEPES-free acid, 0.1% bovine serum albumin, pH 6.5] and, gel-filtered through Sepharose 2B column (Sigma-Aldrich, St. Louis, USA) equilibrated in calcium-free Tyrode buffer, pH 7.2, as mentioned in our work^24^.

### Platelet coagulation assay

The washed platelets (350 × 10^3^/μL) were incubated with various concentrations of DENV2 at 37 °C for 45 min and fixed with 1% paraformaldehyde and washed. The platelets were used as the PS surface, and was incubated with 50 μL of platelet poor plasma and coagulation reagents Kaolin (Diagnostica Stago Inc., Parsippany, USA) including 100 μL of 0.025 M CaCl_2_ at 37 °C for 3 min. The coagulation time was measured using a coagulometer (Diagnostica Stago Inc., Parsippany, USA).

### Immunoblotting

Washed platelets were incubated with various concentrations of DENV2. Platelets pellet were lysed with RIPA buffer in presence of Halt^TM^ protease- phosphatase inhibitor (Thermo Scientific Life Technologies, Omaha, USA). The lysis products were processed for SDS PAGE and immunoblotting for platelet signaling molecule for activation (such as phospho-ERK) and apoptosis (such as caspase 9, cyclophilin D) was done.

### Platelet phagocytosis by monocytes

Human PBMCs were isolated from whole blood using Ficoll gradient density centrifugation. The monocytes were isolated using plate adherence method. The purity of monocytes was checked by flow cytometry using CD14 PE labelled antibody. Washed platelets (350 × 10^3^/μL) were incubated with different MOI of DENV2 for 45 mins at 37 °C. The above reaction mixture (10 μL) was incubated with monocytes (1 × 10^6^ cells/mL). The monocytes were collected at 30 and 60 min time-point and washed to remove surface-bound platelets and used for intracellular staining of platelets. Intracellular staining was performed using a BD Cytofix/Cytoperm fixation and permeabilization kit (BD Biosciences). The cells were fix and resuspended in permeablization buffer as per manufacturer’s protocol and labelled with CD42b APC (BD Biosciences, San Jose, USA) and analyzed using flow cytometry as mentioned^36^.

### Parallel flow chamber

#### Platelet thrombus formation assay

The whole blood collected from healthy individuals was perfused over the petri plate confluent with human umbilical vein endothelial cells (HUVEC) from ATCC, USA as mentioned in our recent work^25^. HUVEC monolayer was activated with 25 μM histamine (Sigma, USA) before fitting into parallel flow chamber. Whole blood was incubated with DENV2 for 1 hr and mepacrine (0.2 M, Sigma-Aldrich, USA; used for labeling platelets) for 5 min before perfusion. A syringe pump (Harvard Apparatus Inc., USA) was connected to the outlet port that drew blood through the chamber at the shear stress of 2.5 dyne/cm^2^. The flow chamber was mounted onto a Nikon Eclipse Ti-E inverted stage microscope (Nikon, Japan) equipped with a high-speed digital camera. The fluorescence image and movie was recorded at magnification 40X and analyzed using NIS-Elements version 4.2 software. The platelet thrombus size as well as number of VWF-platelet strings was measured.

### Statistical analysis

Experimental values were presented as mean ± standard error. The Mann-Whitney Test, Student’s t-test and one-way ANOVA were used for data analysis, and a p-value less than 0.05 was considered to be statistically significant. Spearman correlation and linear regression analysis was performed with confidence interval 95% using Sigma plot 12.0.

## Additional Information

**How to cite this article**: Ojha, A. *et al*. Platelet activation determines the severity of thrombocytopenia in dengue infection. *Sci. Rep.* **7**, 41697; doi: 10.1038/srep41697 (2017).

**Publisher's note:** Springer Nature remains neutral with regard to jurisdictional claims in published maps and institutional affiliations.

## Supplementary Material

Supplementary Dataset 1

Supplementary Dataset 2
