# Supplementary material for: Platelet activation determines the severity of thrombocytopenia in dengue infection
Source: Sci Rep. 2017 Jan 31;7:41697. doi: 10.1038/srep41697 (PMC5282509; doi:10.1038/srep41697)
Supplement: Supplementary Dataset 1 [file srep41697-s1.doc]

**Platelet activation determines the severity of thrombocytopenia in dengue infection.**

Amrita Ojha^1,2^, Dipika Nandi^1^, Harish Batra^3^, Rashi Singhal^1,2^, Gowtham K Annarapu^1,2^, Sankar Bhattacharyya^3^, Tulika Seth^4^, Lalit Dar^5^, Guruprasad R Medigeshi^3^, Sudhanshu Vrati^3^, Naval K Vikram^4^ and Prasenjit Guchhait^1,*^

^1^Disease Biology Laboratory, Regional Centre for Biotechnology, National Capital Region Biotech Science Cluster, Faridabad, India. ^2^Department of Biotechnology, Manipal University, Karnataka, India. ^3^Vaccine and Infectious Disease Research Center, Translational Health Science and Technology Institute, National Capital Region Biotech Science Cluster, Faridabad, India. Department of ^4^Medicine,^5^Microbiology, All India Institute of Medical Sciences, New Delhi, India.

^*^Correspondence: Prasenjit Guchhait at [prasenjit@rcb.res.in.](mailto:prasenjit@rcb.res.in)  Phone: (91)129-2848821.


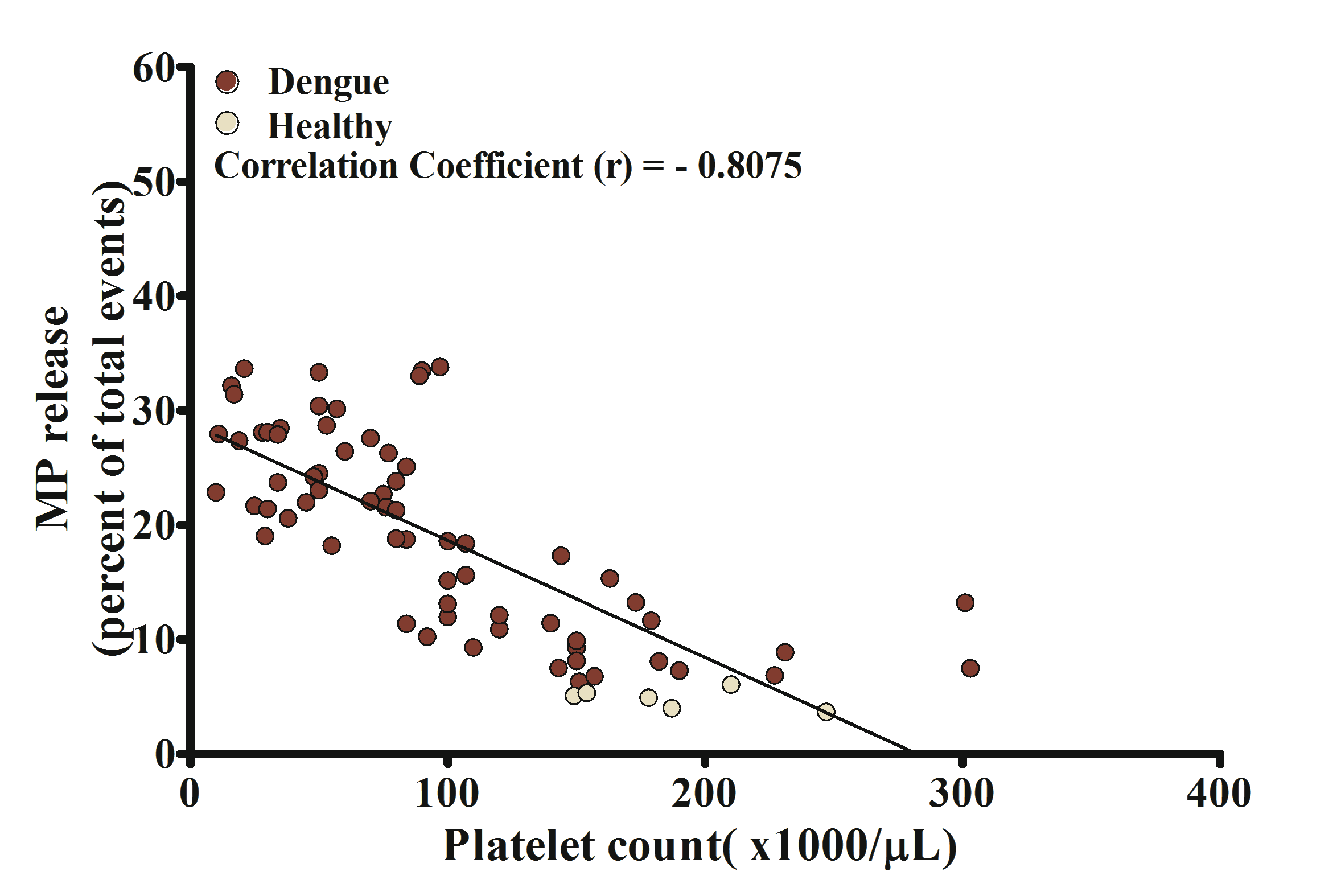


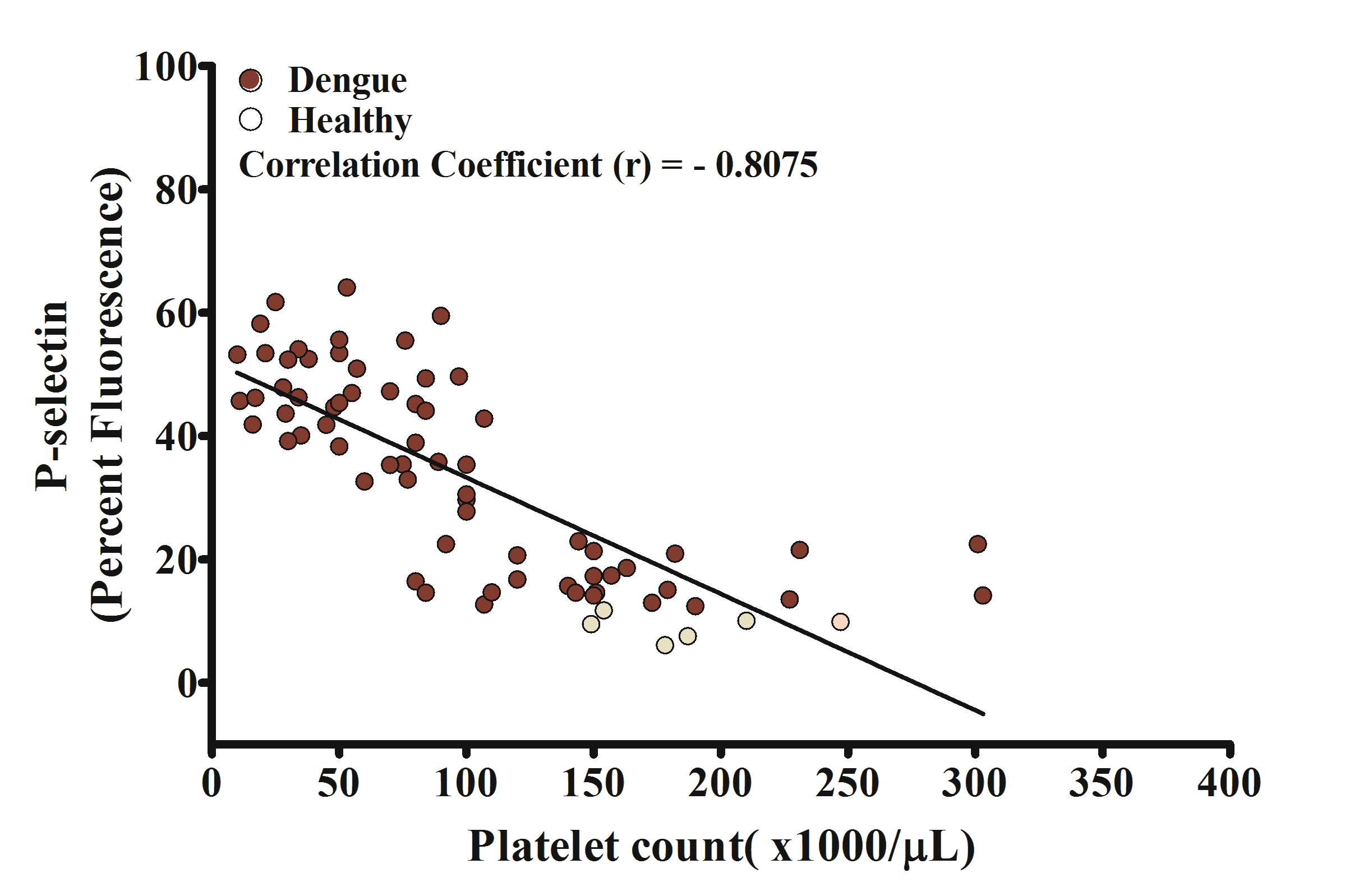

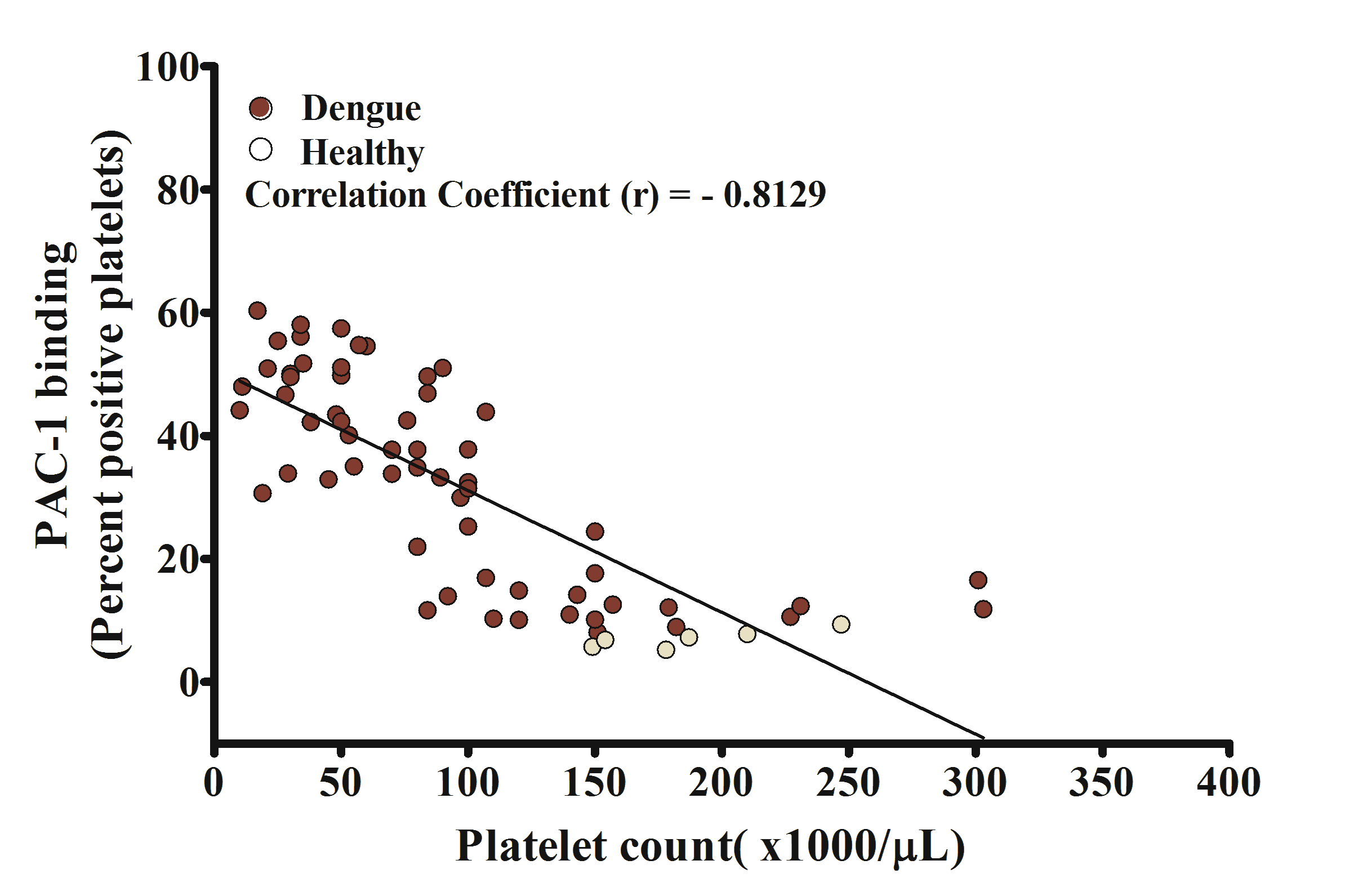

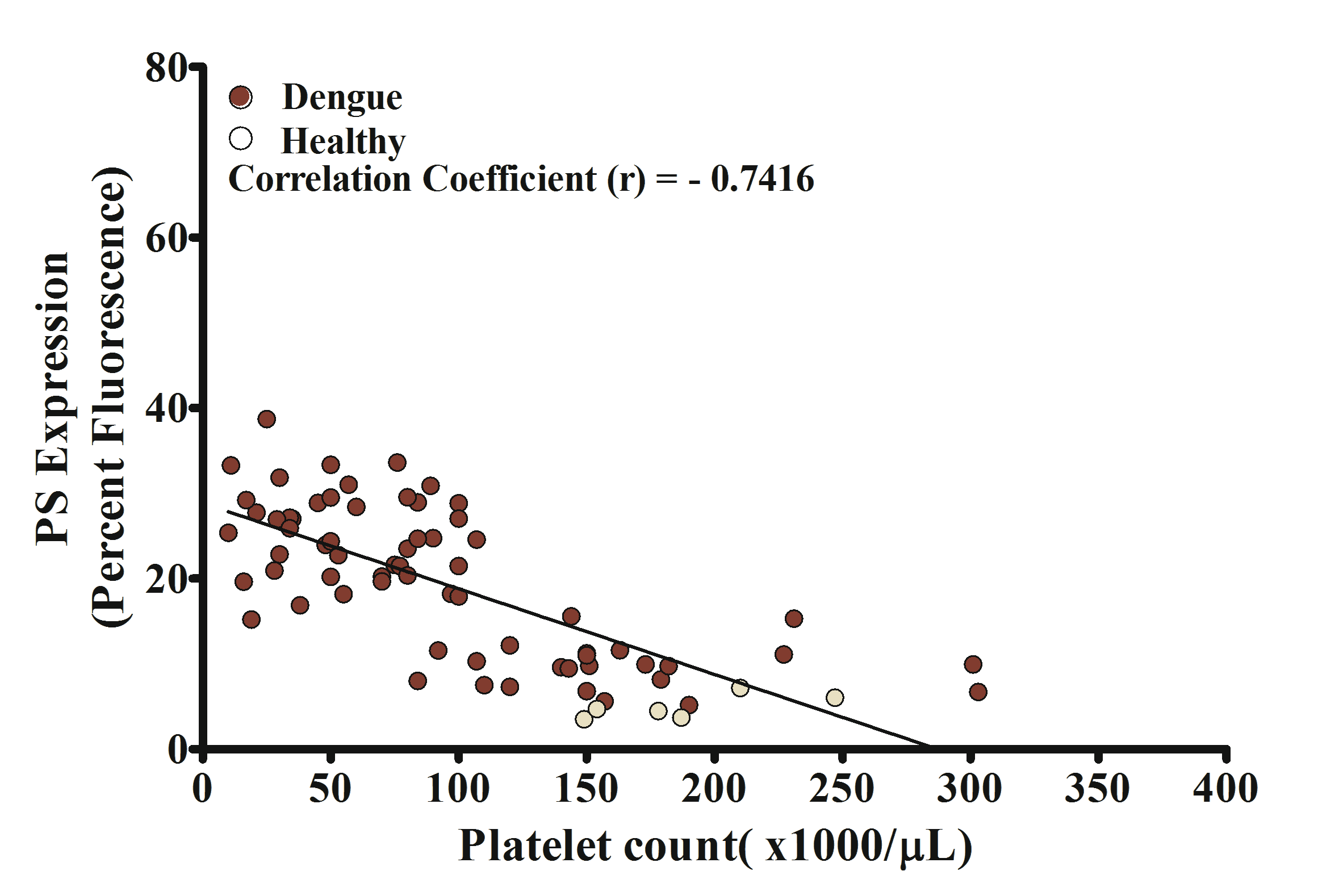


**A**

**B**

**C**

**D**

**Suppl. Fig. 1.** Correlation analysis between platelet counts (as in Fig. 1A) and P-selectin expression on platelets (mentioned in Fig. 1B) **(A)**, PAC-1 binding to platelets (mentioned in Fig. 1C) **(B)**, PS expression on platelets (mentioned in Fig. 1D) **(C)** and platelet-MPs in plasma (mentioned in Fig. 1E) **(D)**.


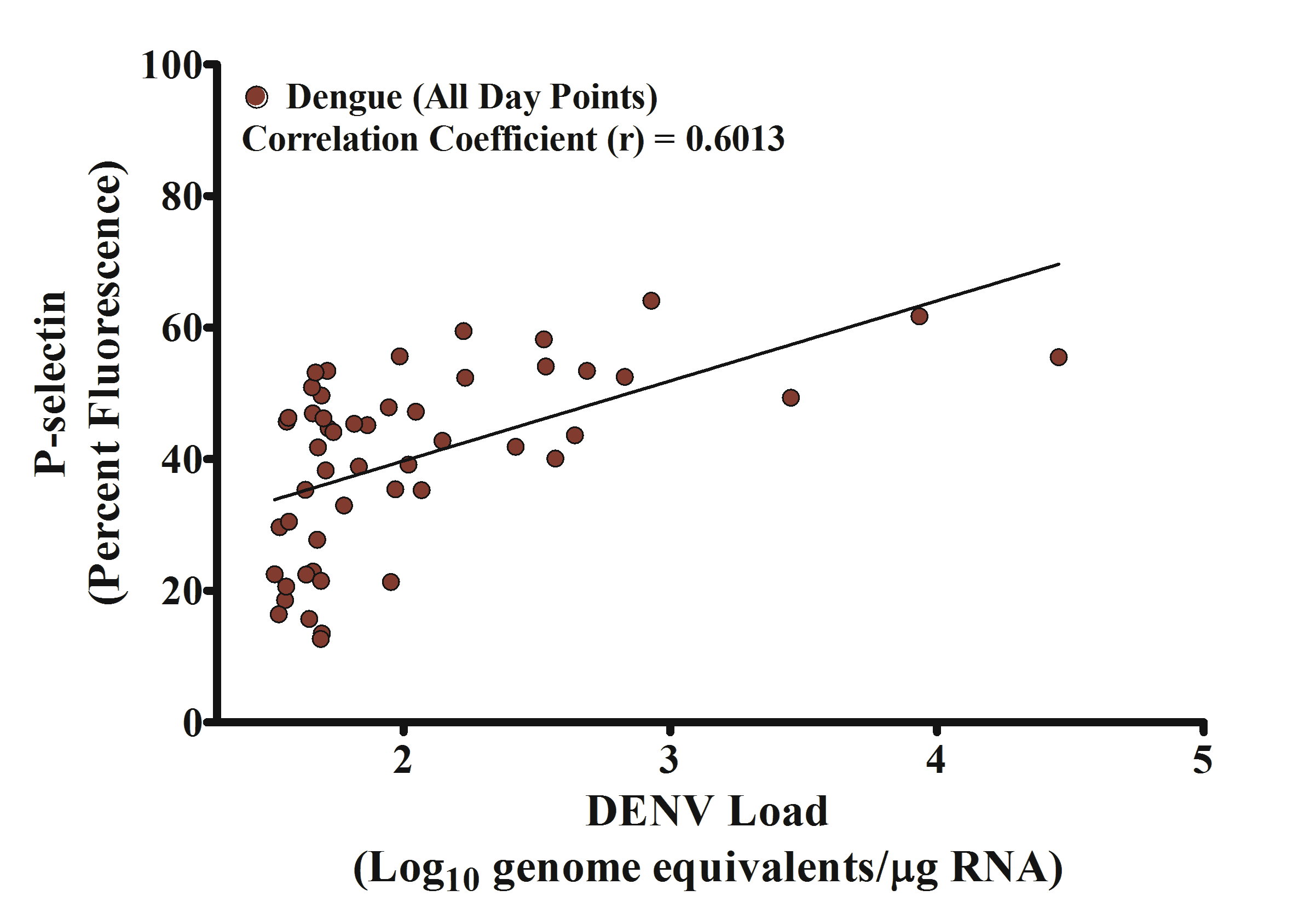

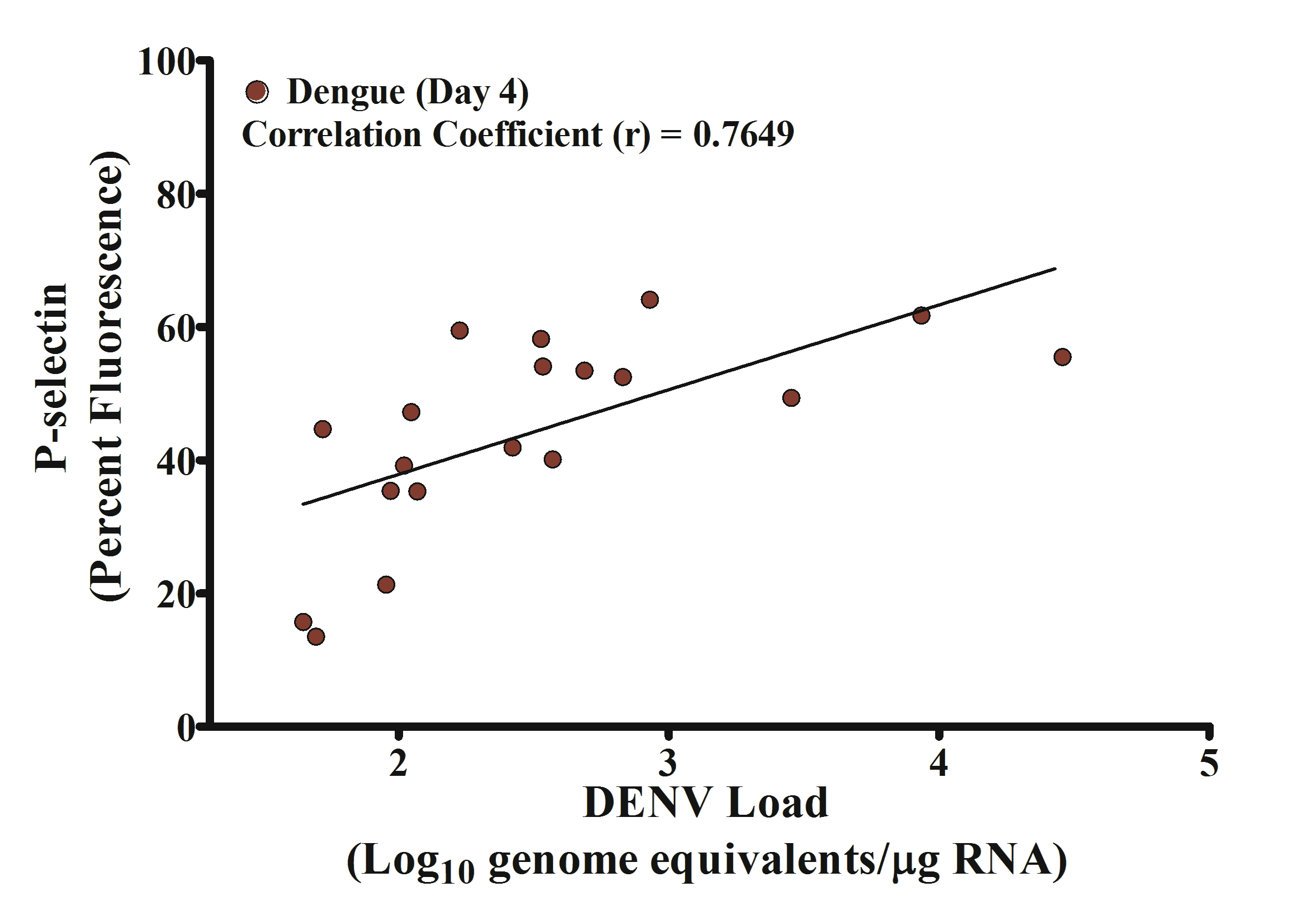

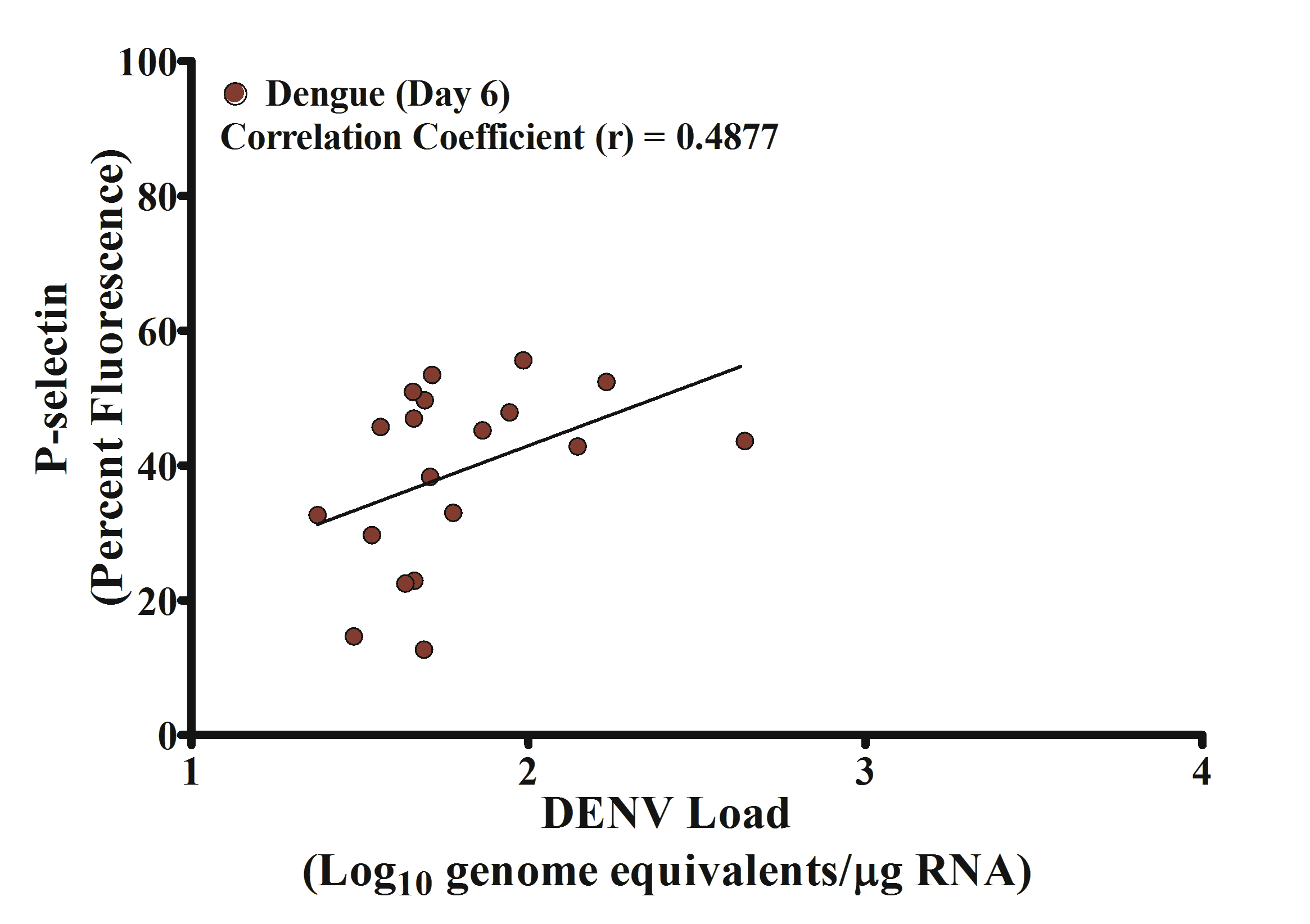

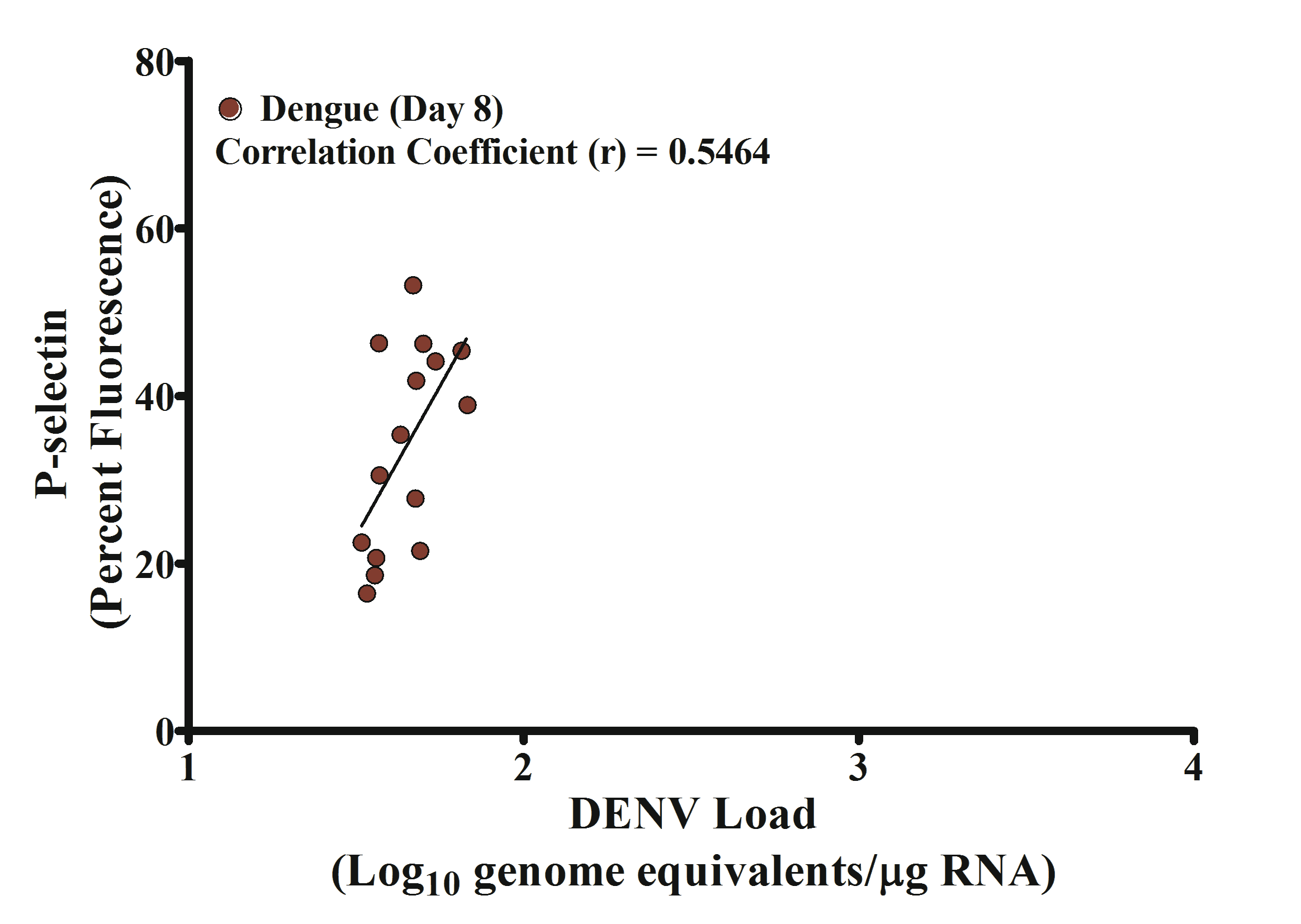


**A**

**Aa**

**Ab**

**Ac**


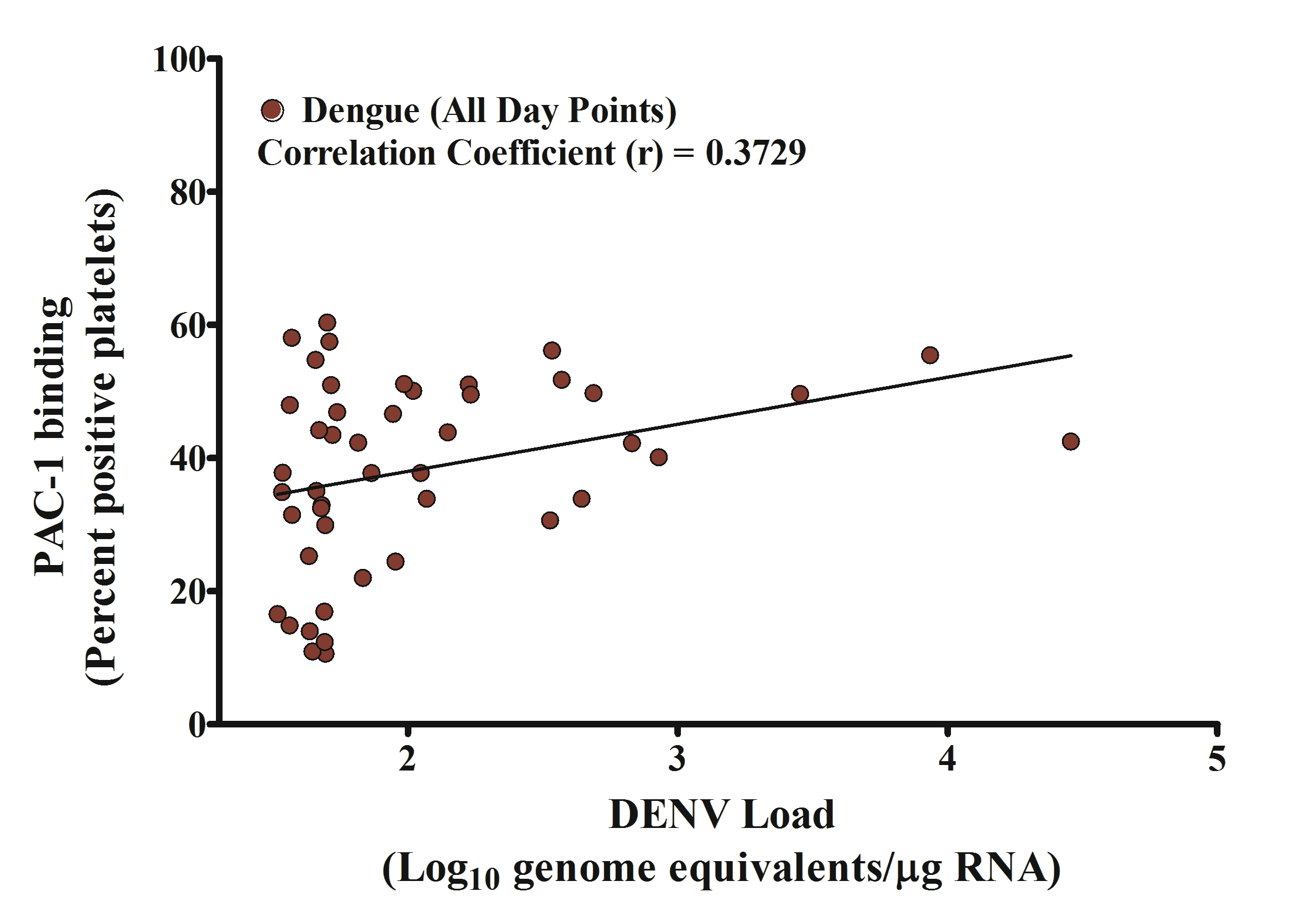

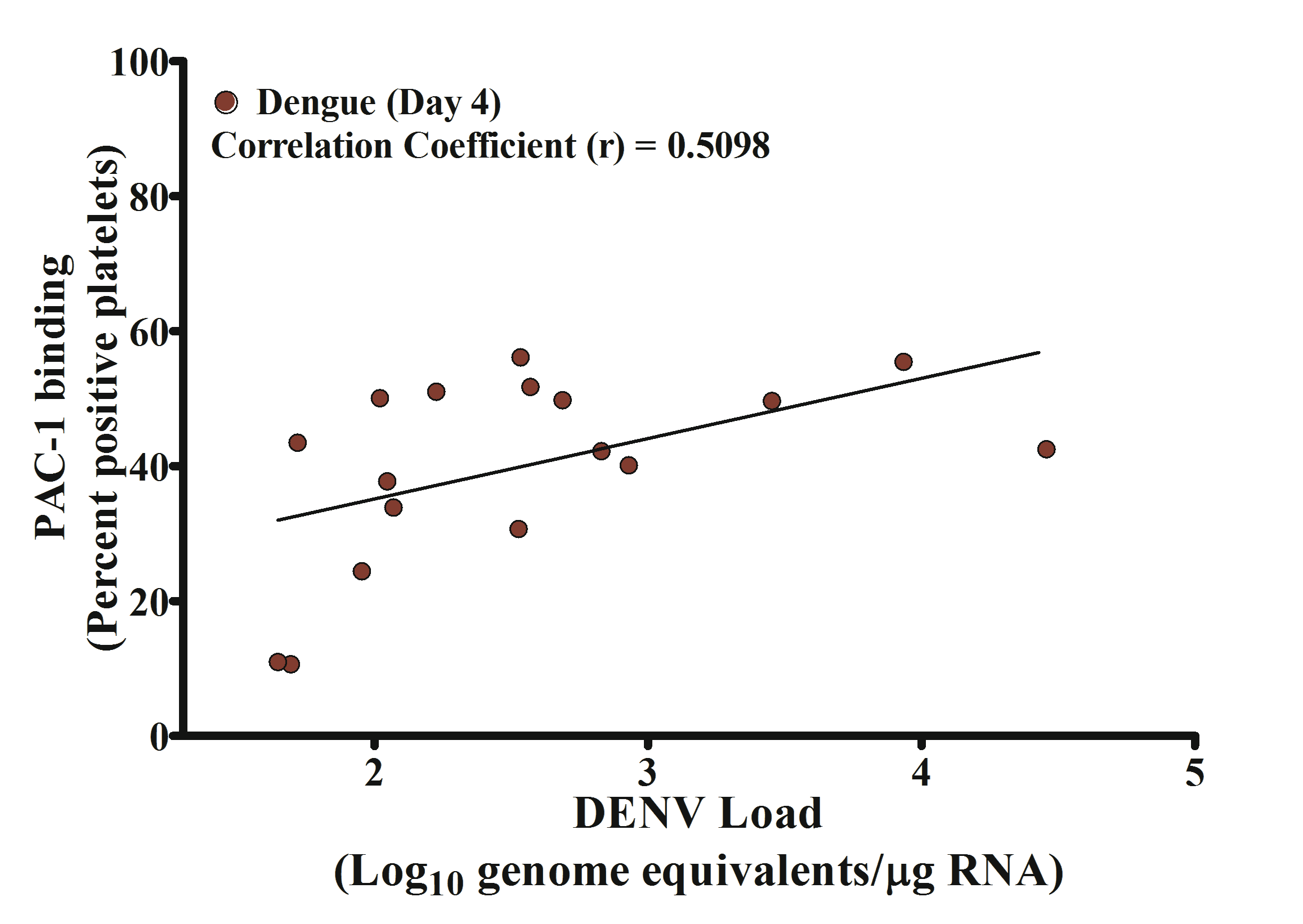


**B**

**Ba**


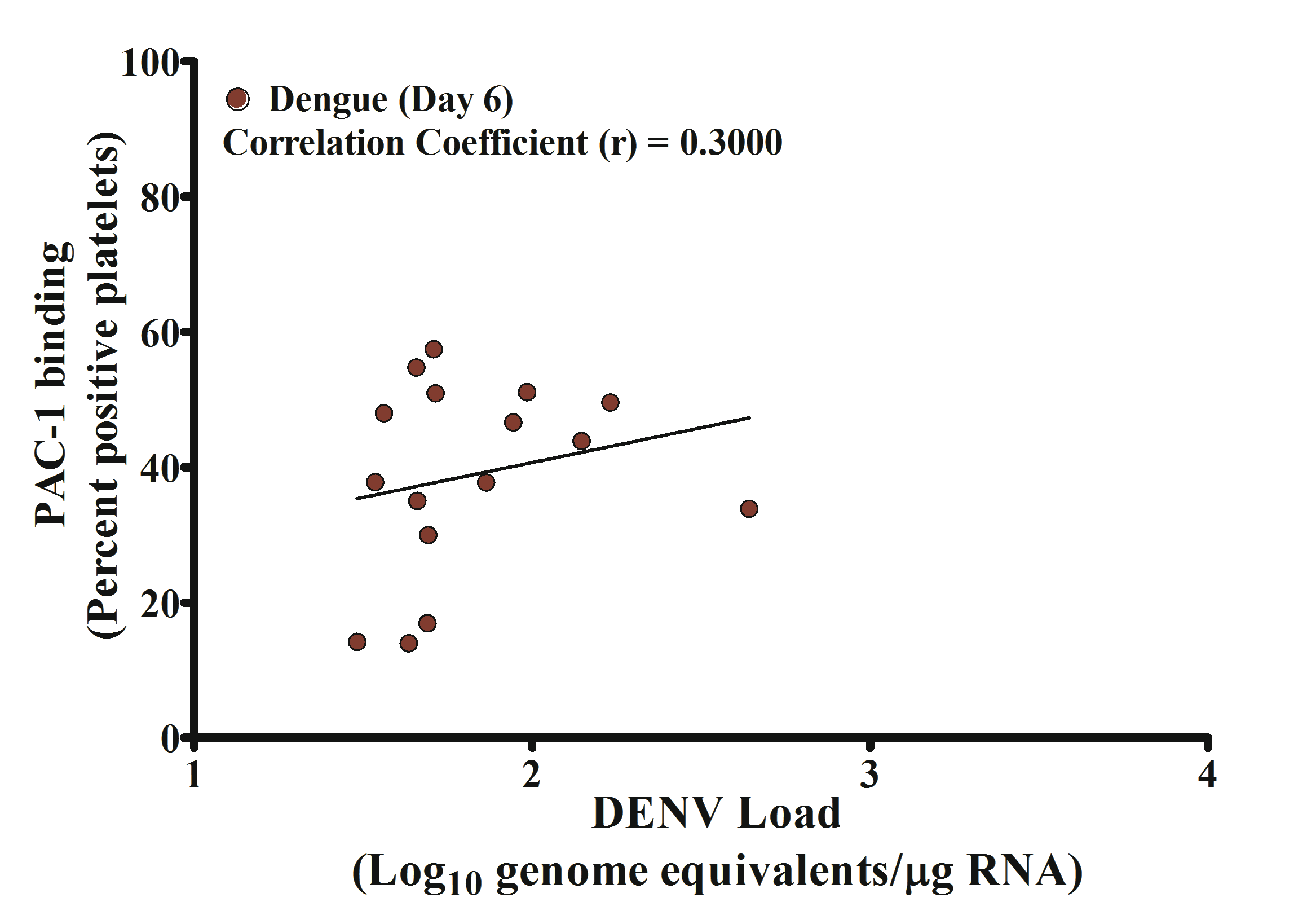

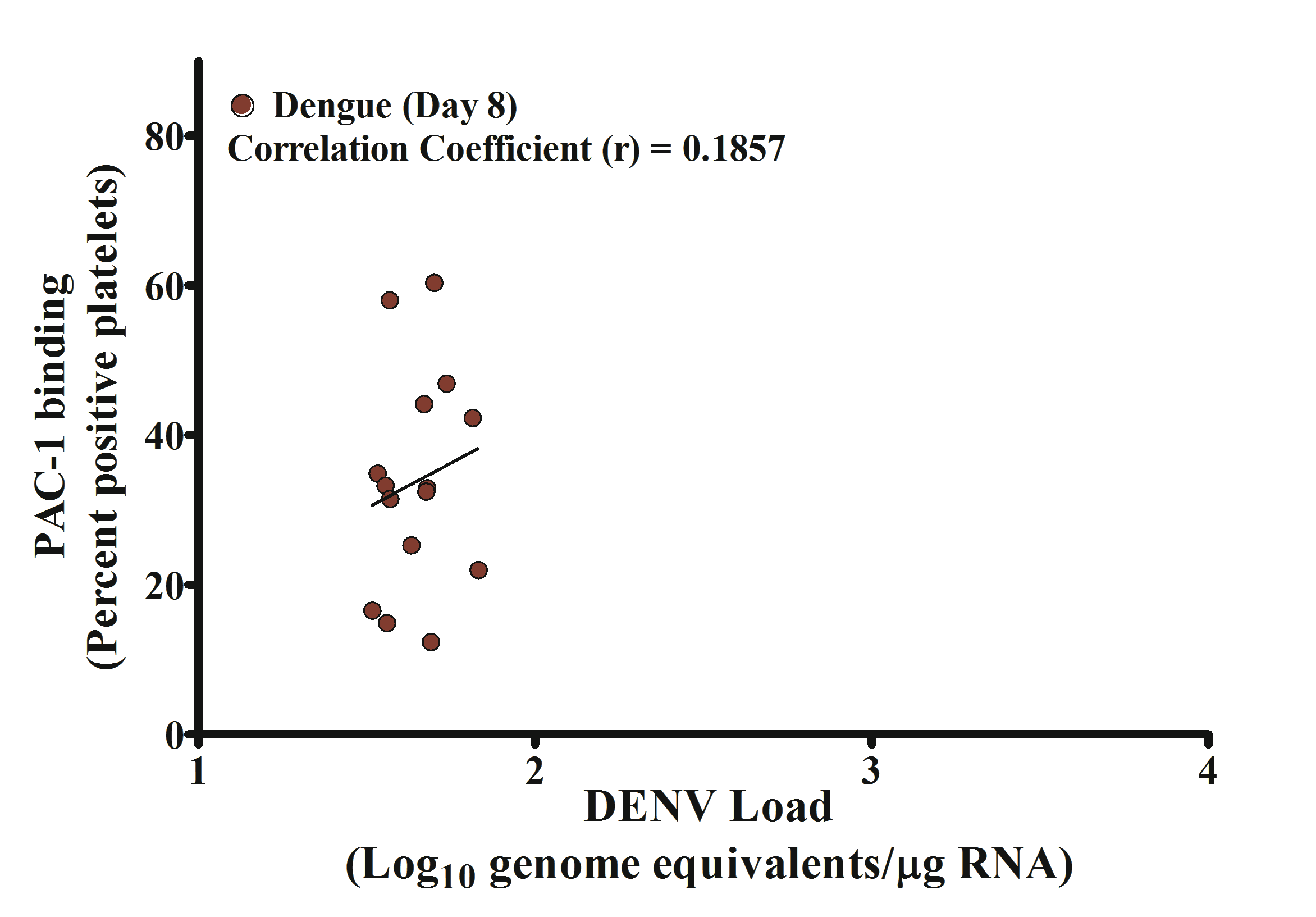


**Bb**

**Bc**


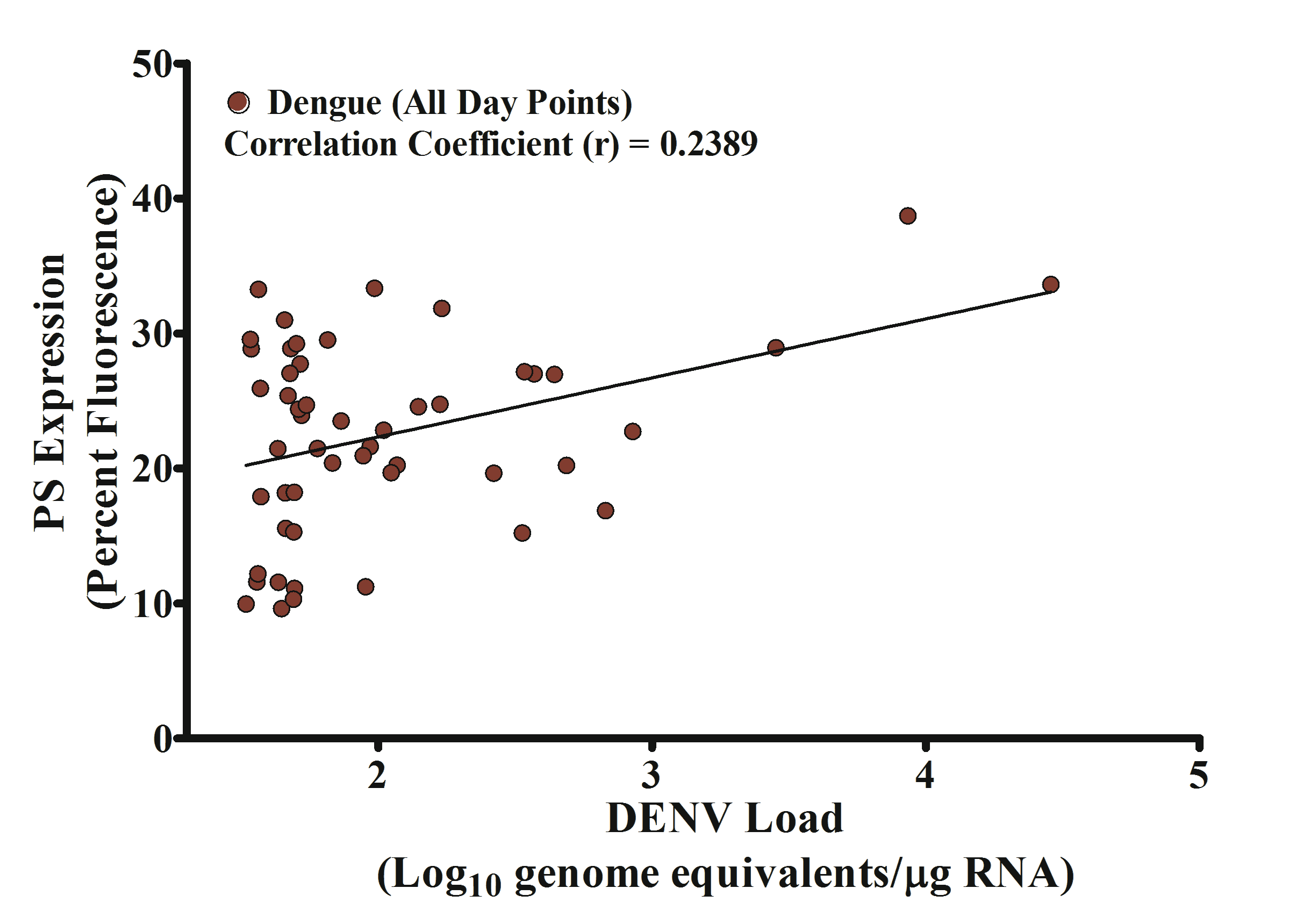

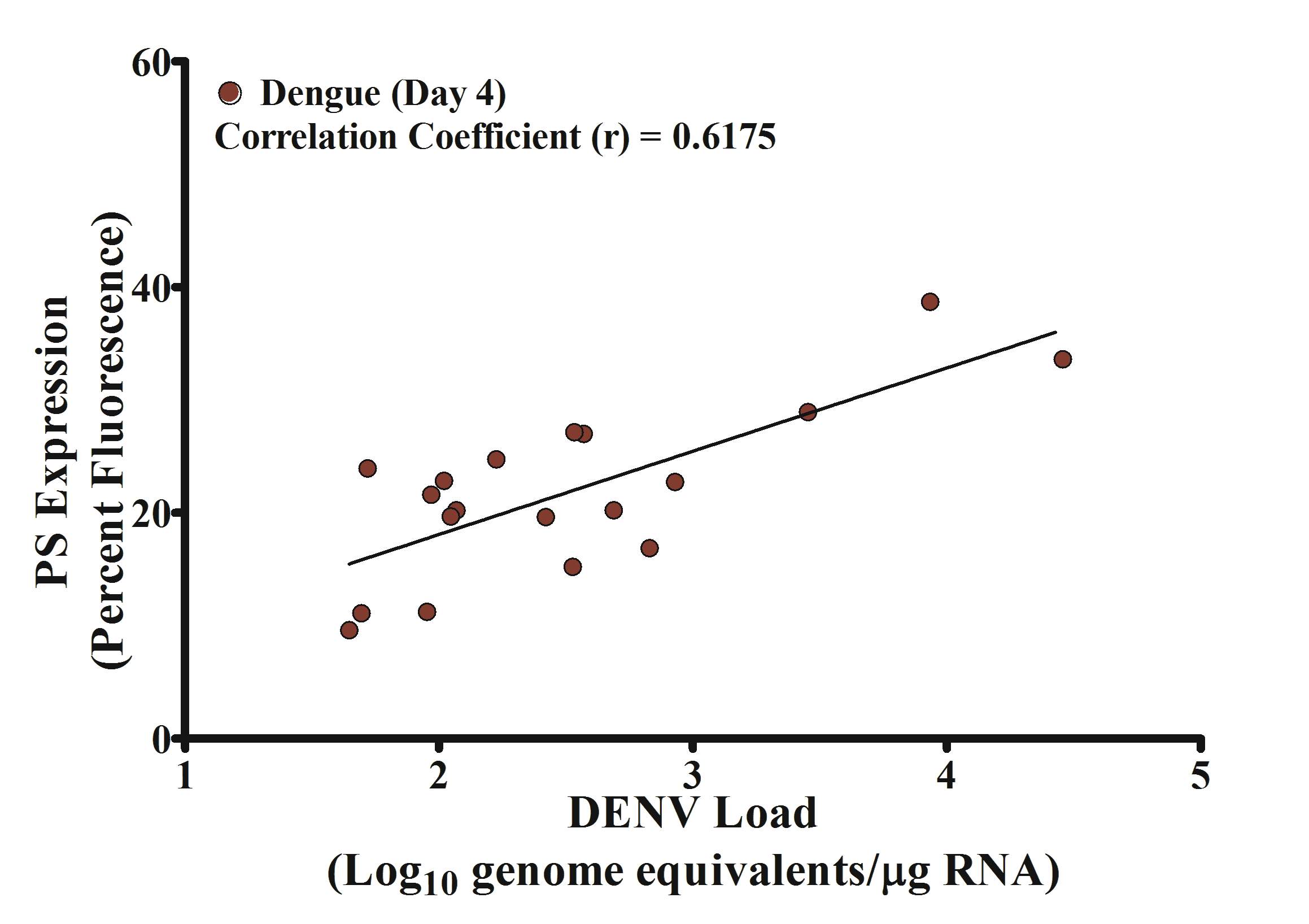

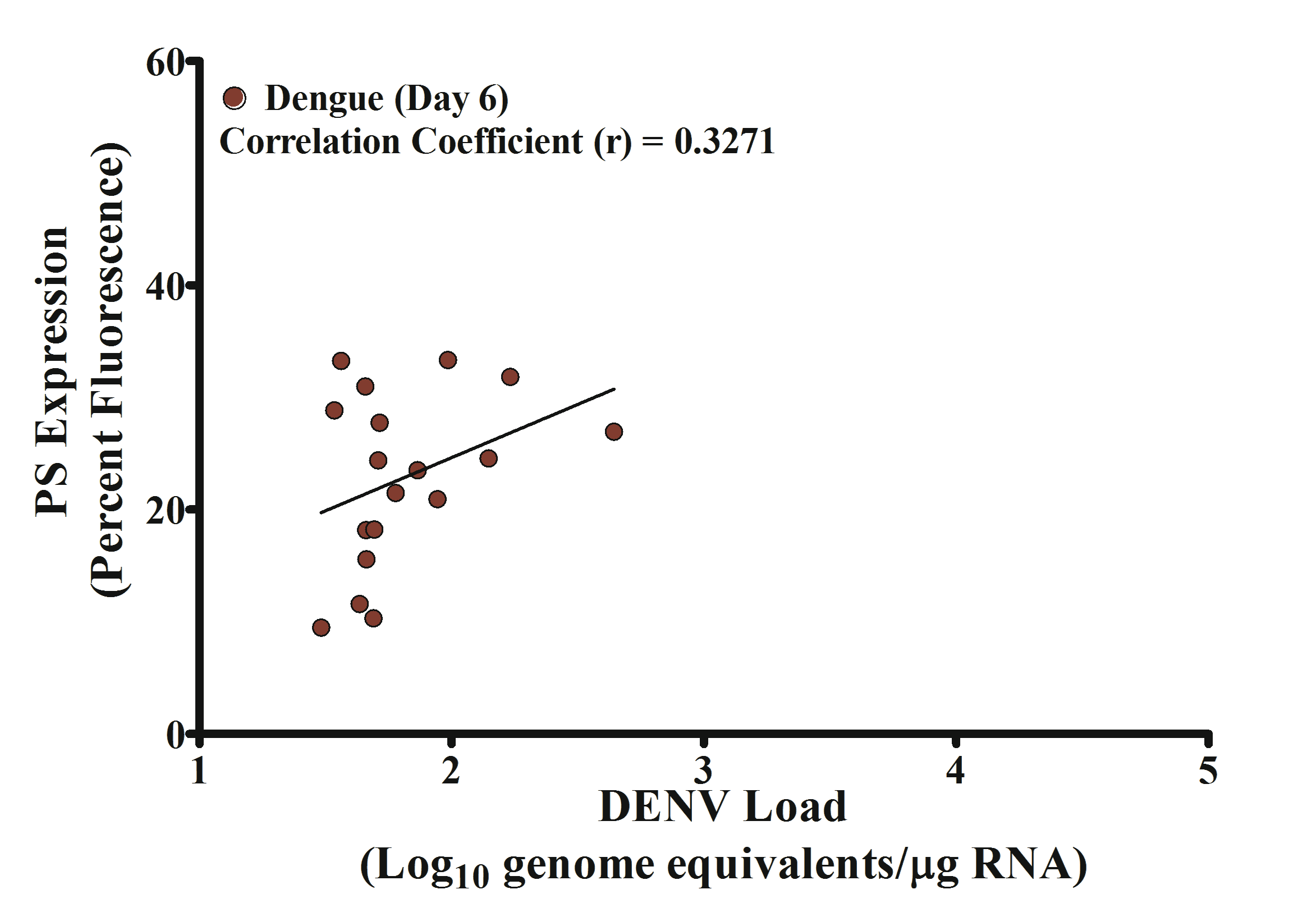

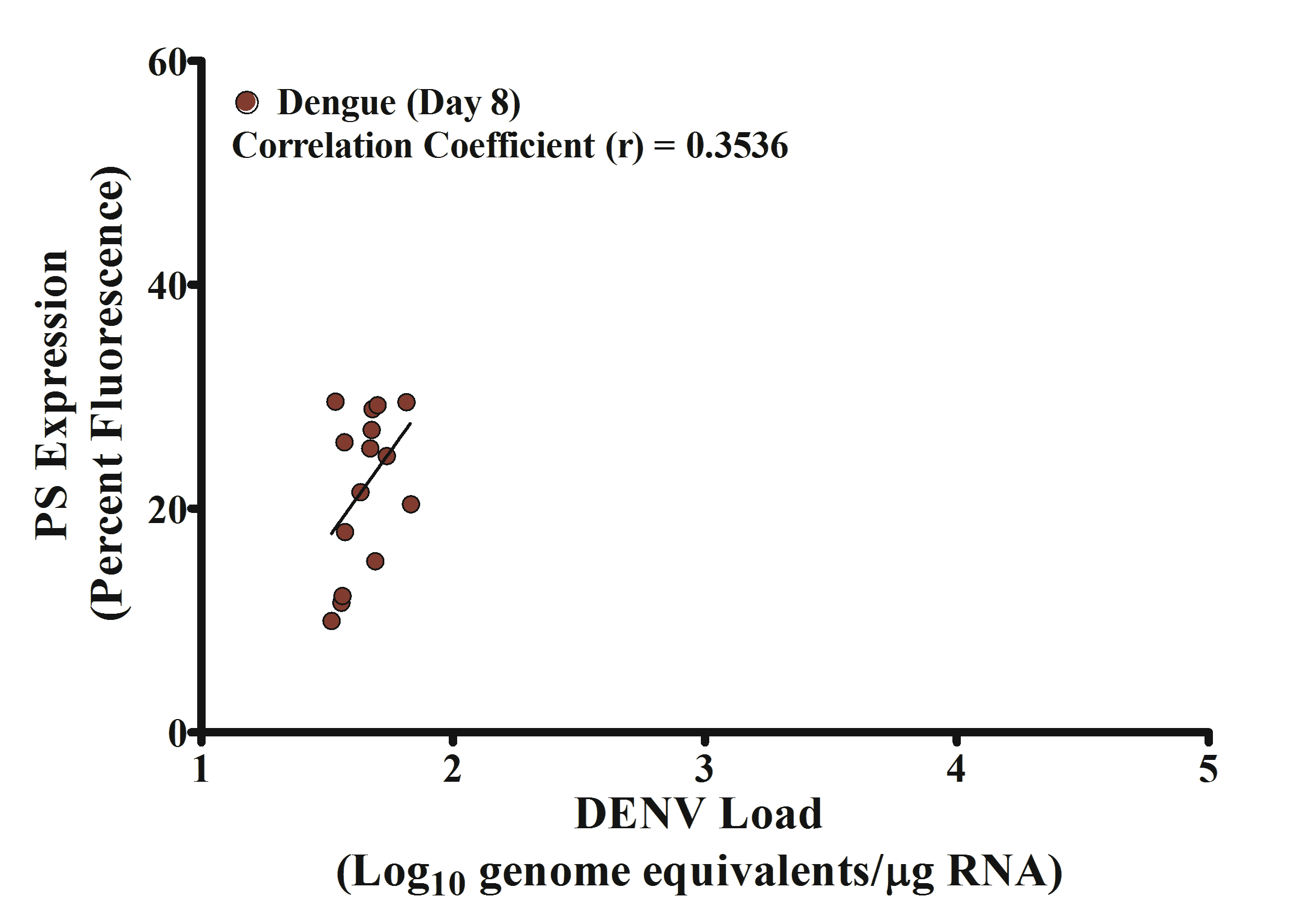


**C**

**Ca**

**Cb**

**Cc**


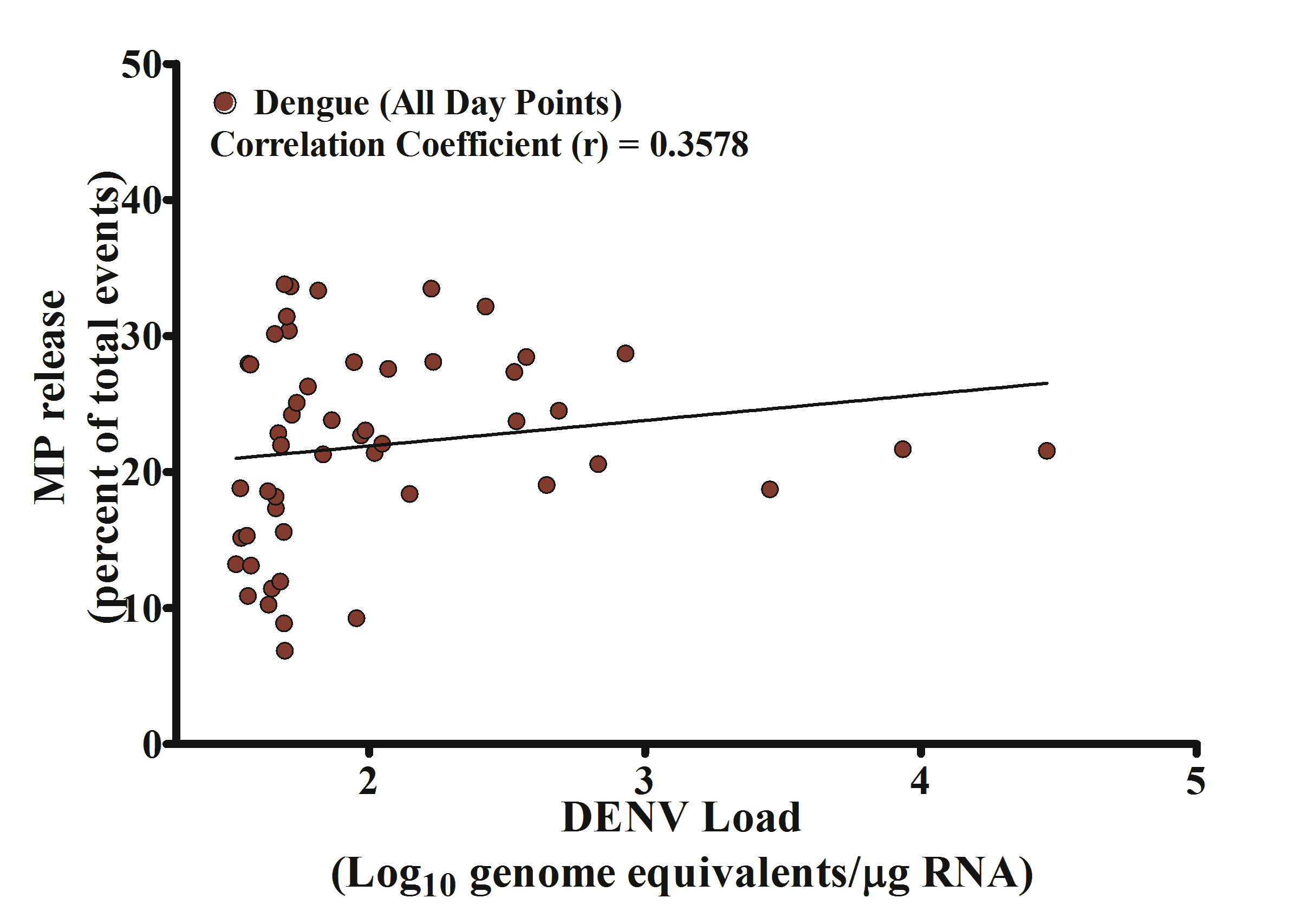

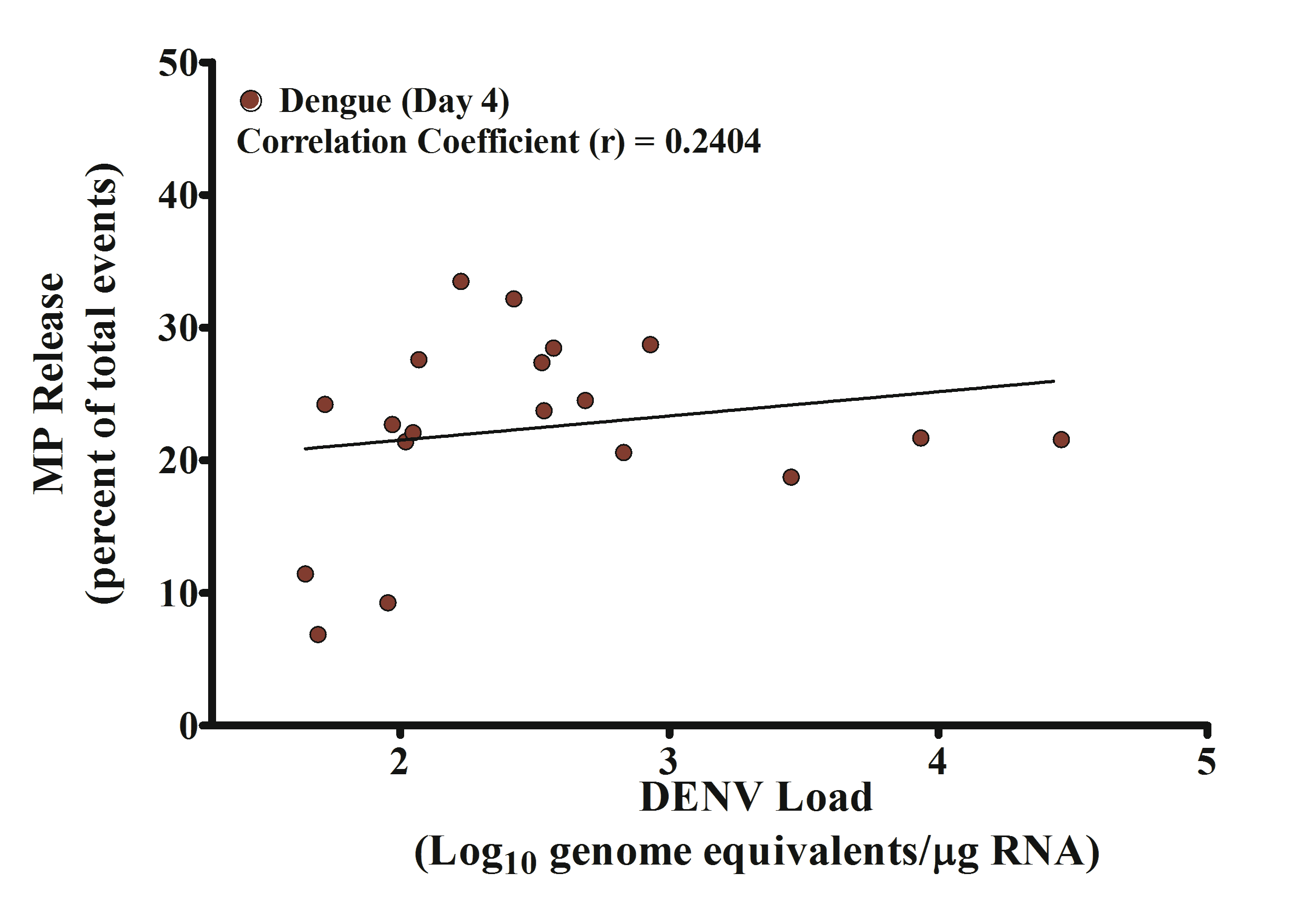

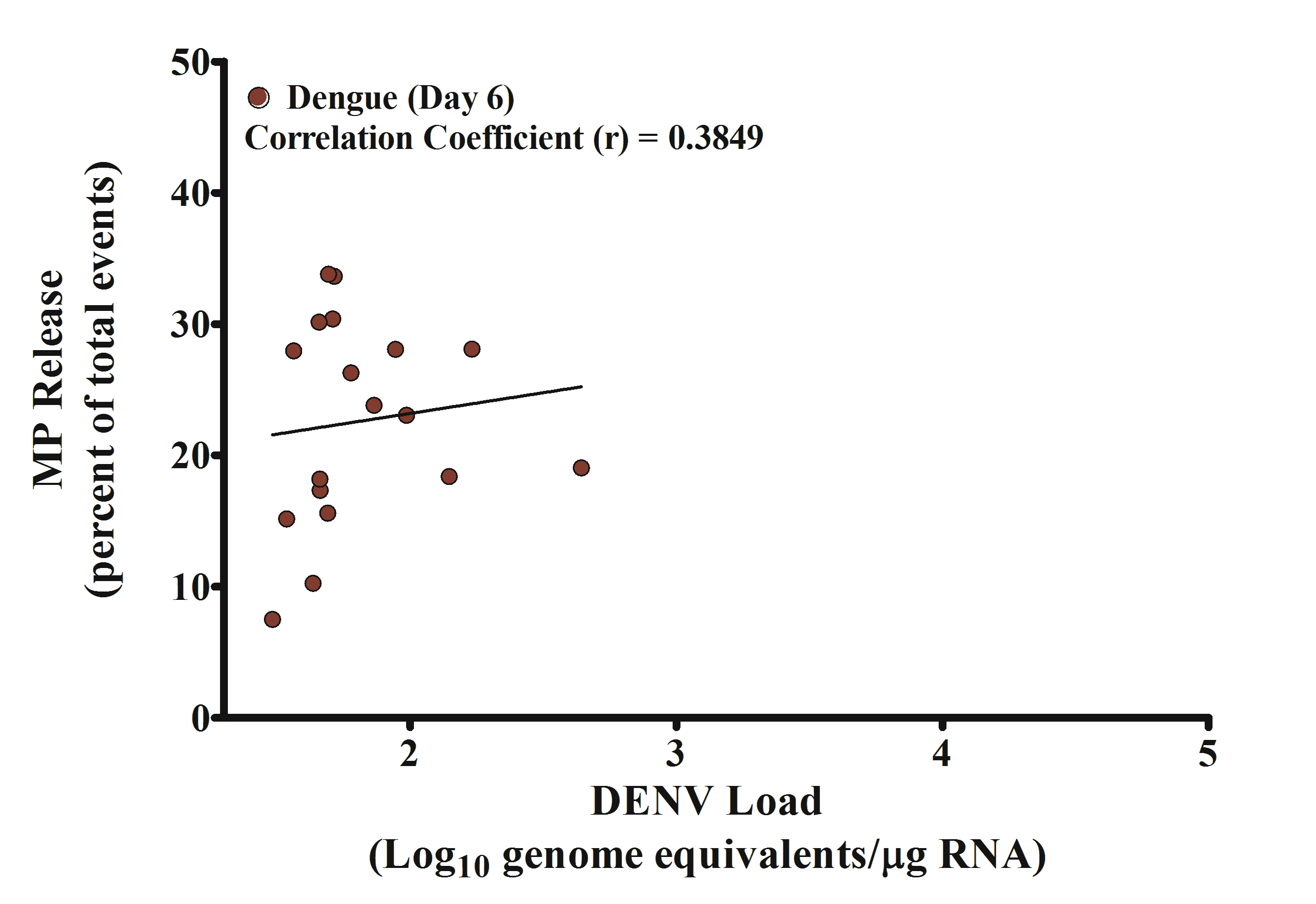

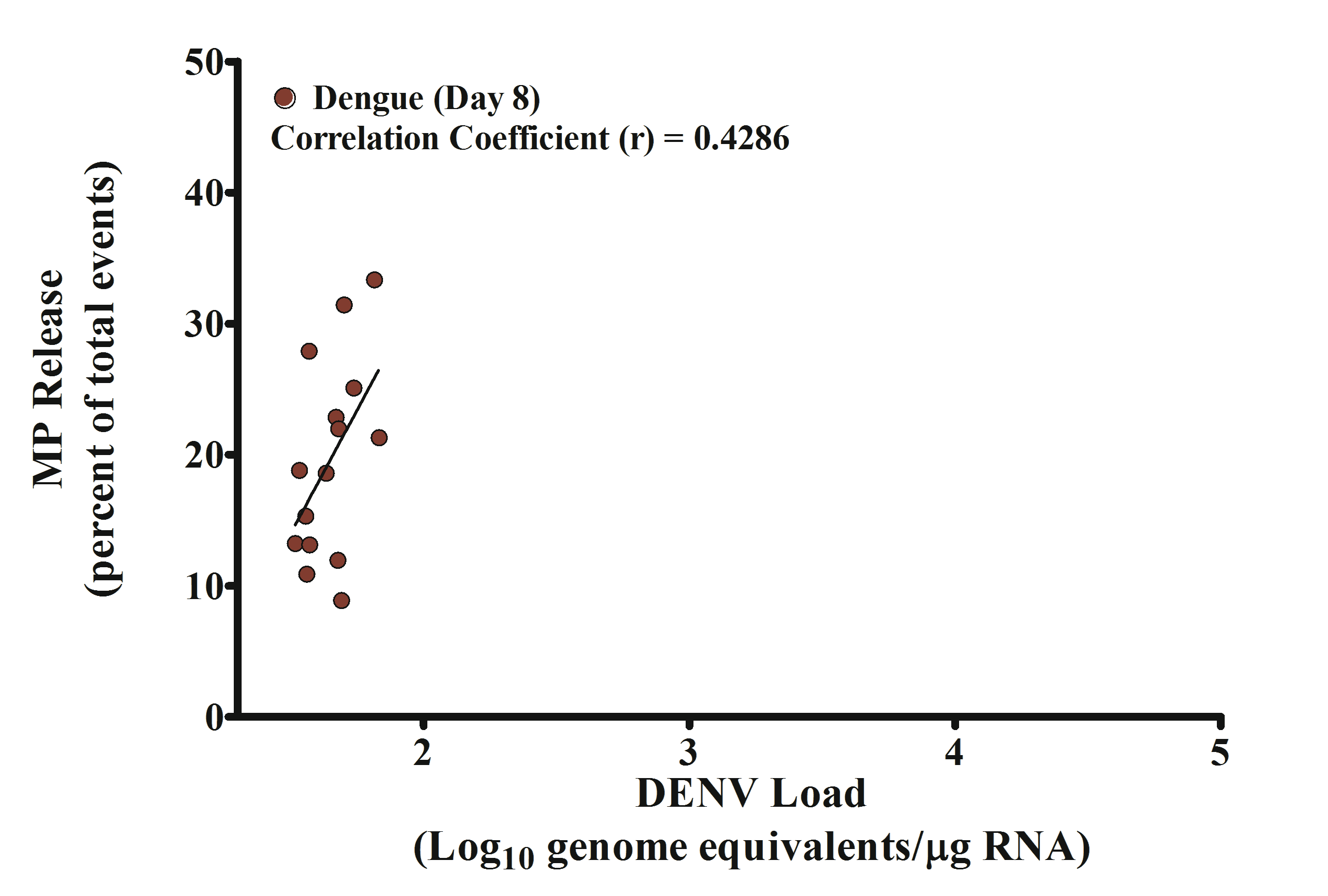


**D**

**Da**

**Db**

**Dc**

**Suppl. Fig. 2.** Correlation analysis between copy number of DENV genome in platelet pellet (as in Fig. 2) and P-selectin expression on platelets at all day points as well as at Day 4, Day 6 and Day 8 separately (mentioned in Fig. 1B) **(2A, 2Aa, 2Ab, 2Ac)**. The PAC-1 binding to platelets (mentioned in Fig. 1C) **(2B, 2Ba, 2Bb, 2Bc, 2Bc)**, and PS expression on platelets (mentioned in Fig. 1D) **(2C, 2Ca, 2Cb, 2Cc,)** and platelet- MPs in plasma (mentioned in Fig. 1E) **(2D, 2Da, 2Db, 2Dc, 2Dd)**.


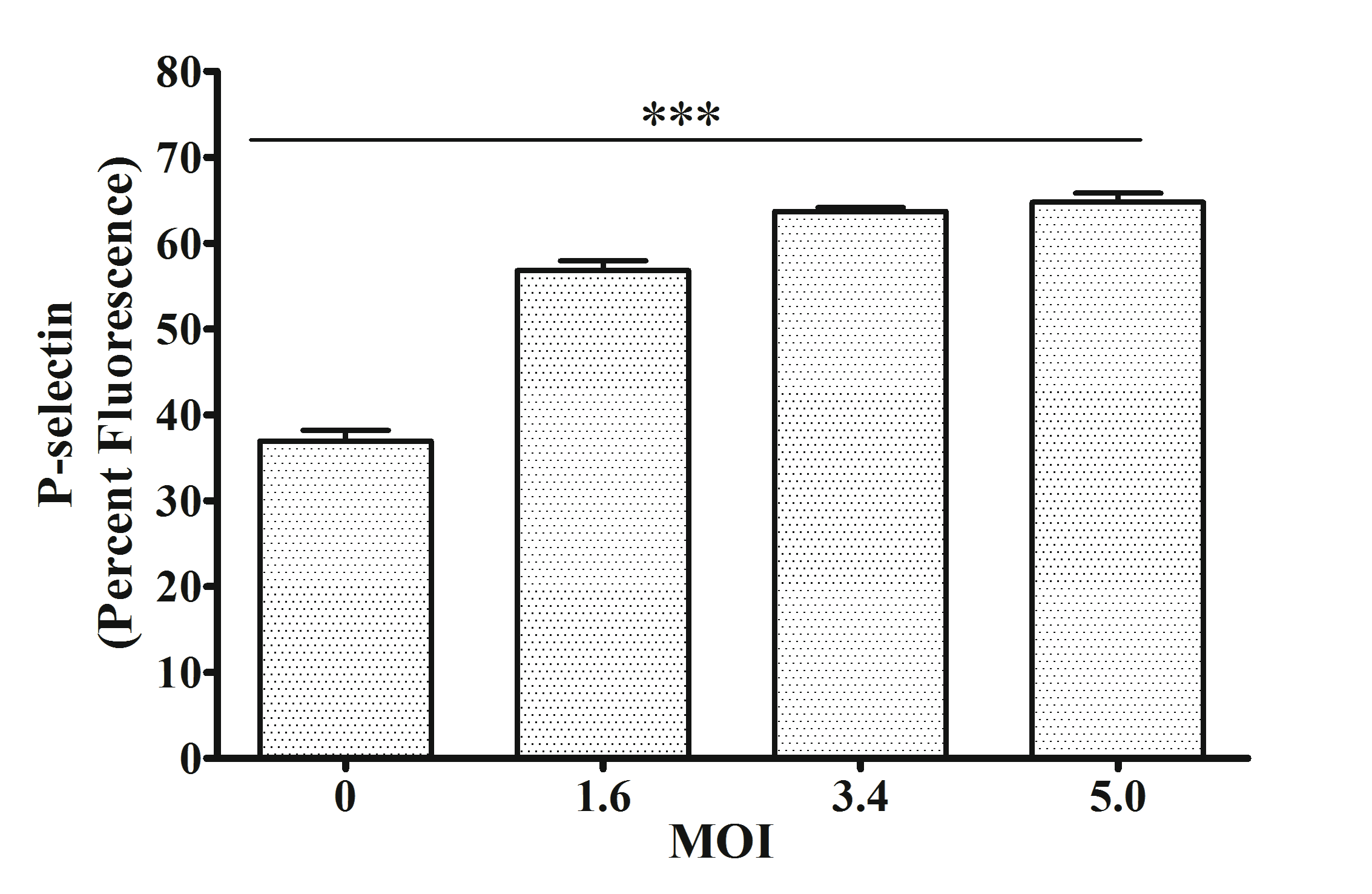

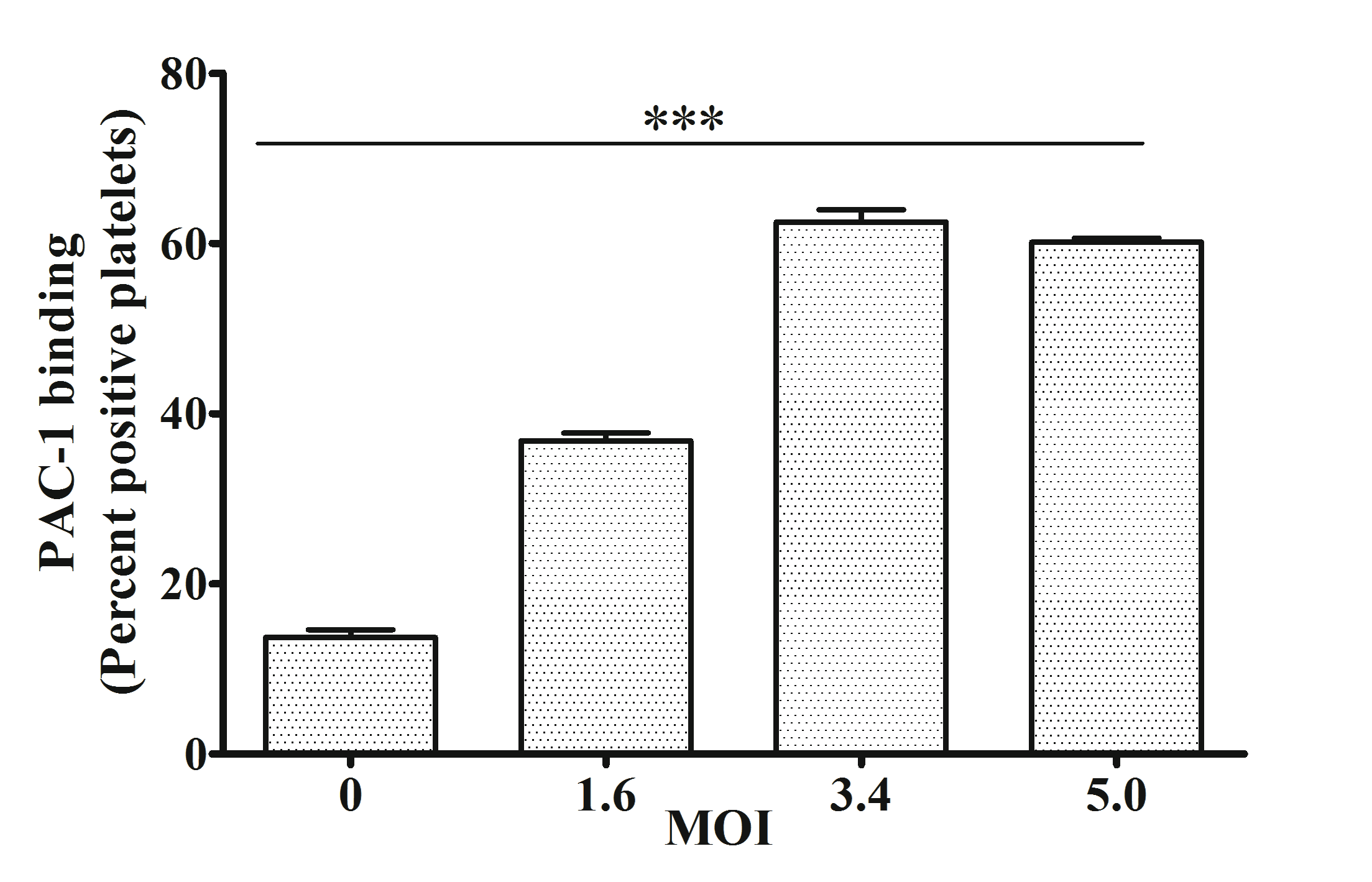

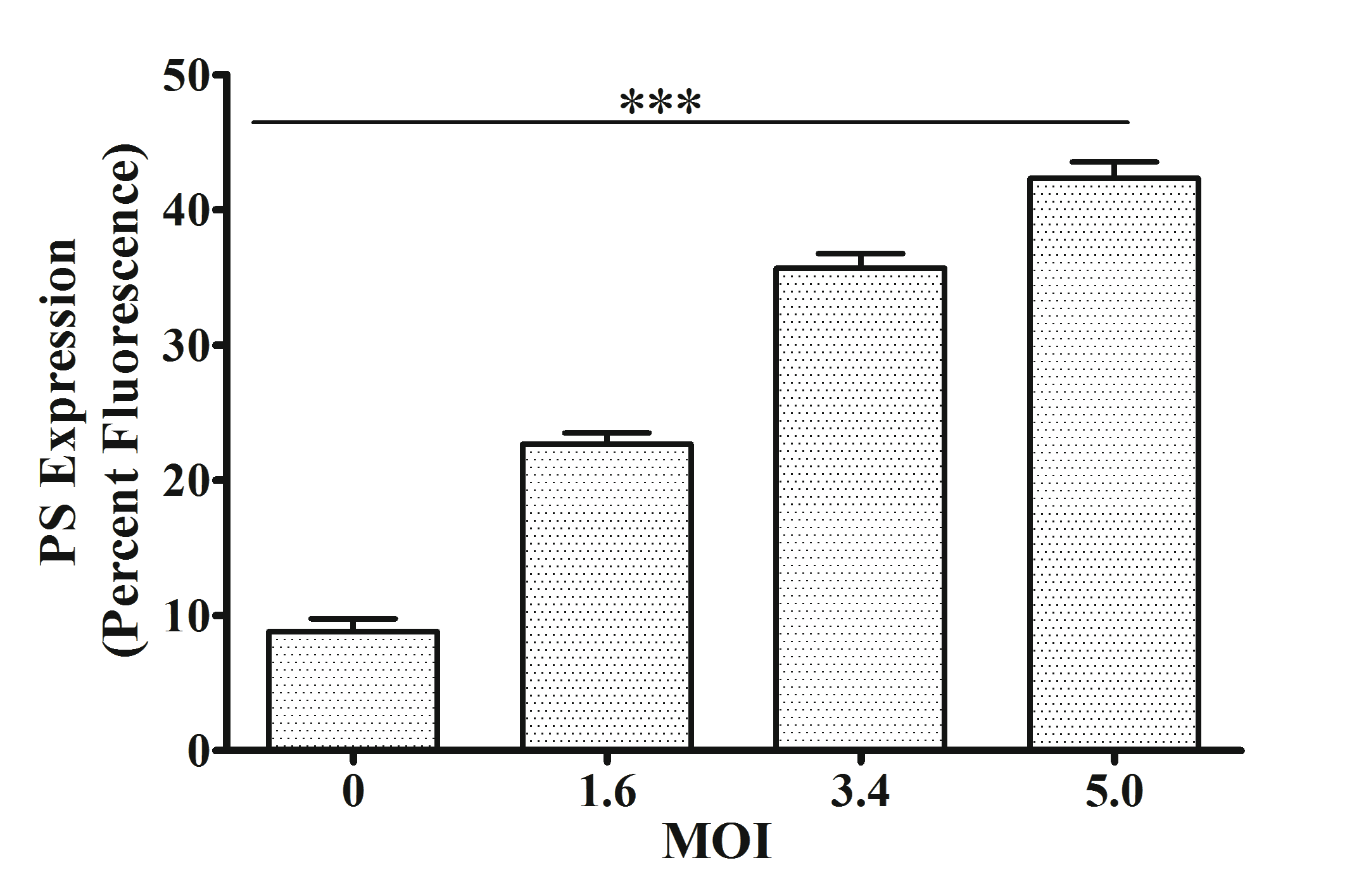

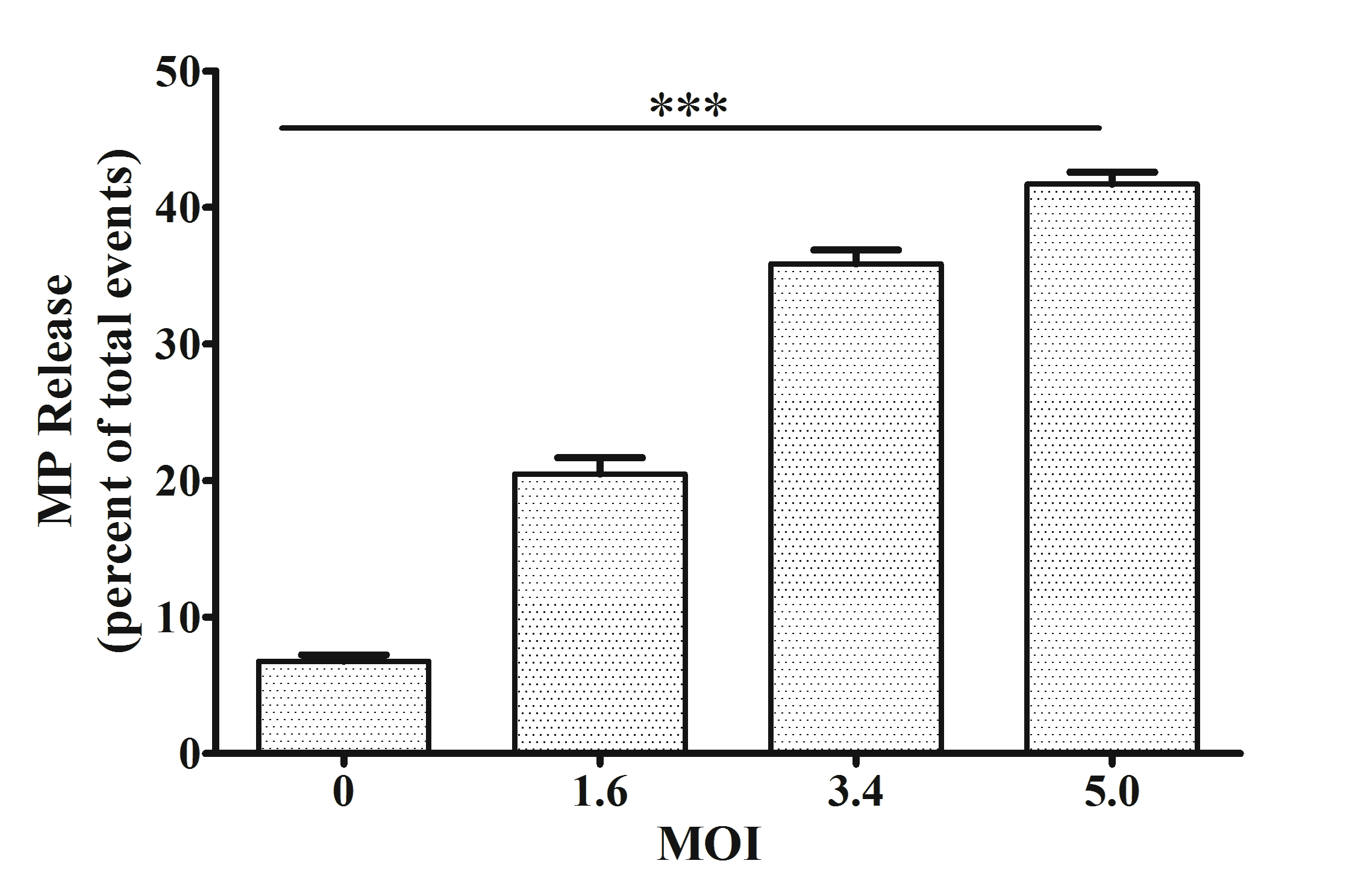


**A**

**B**

**C**

**D**


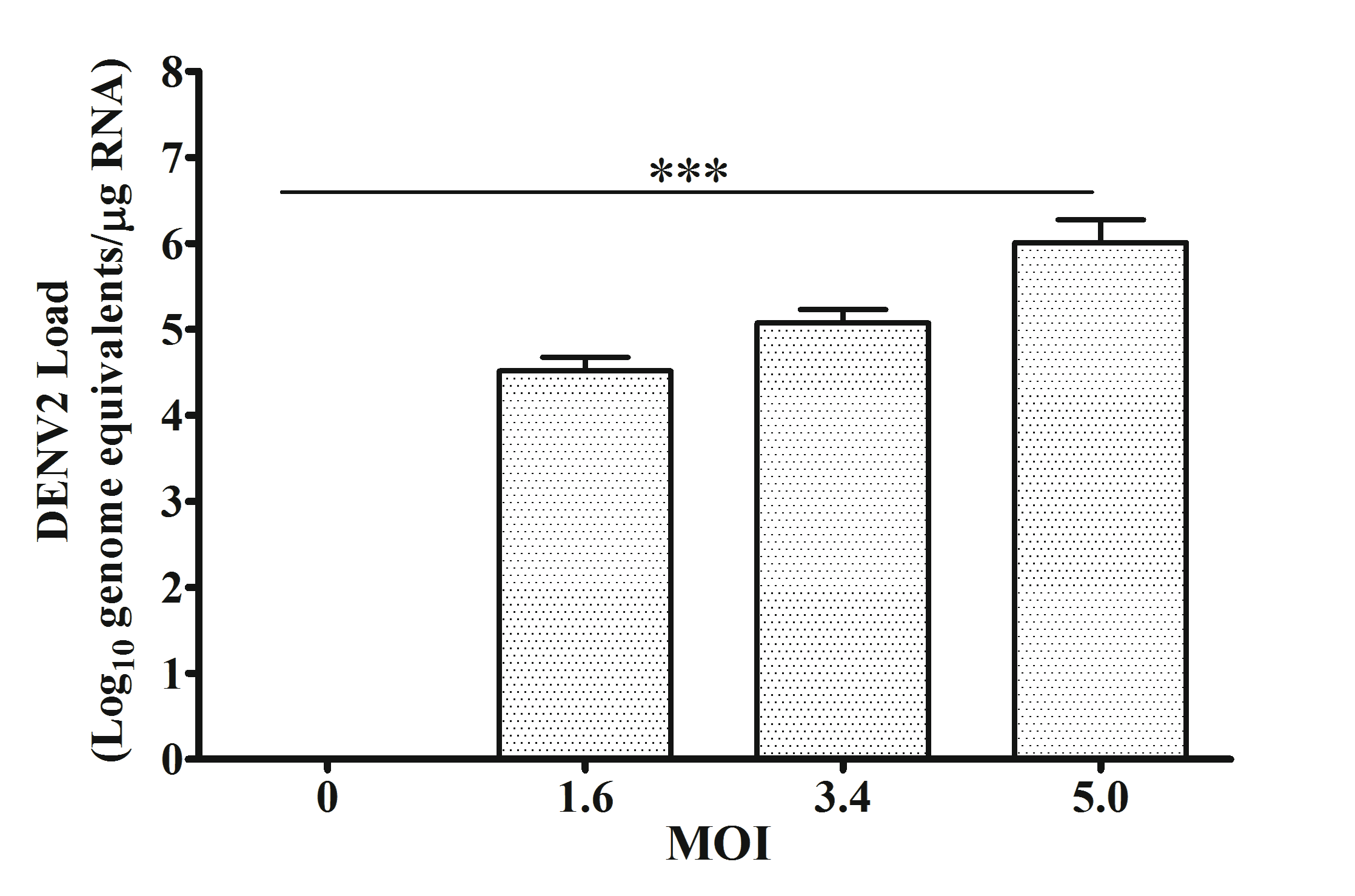


**E**

**Suppl. Fig.3. Platelet activation mediated by DENV2 *in vitro*.** Washed platelets were incubated with different MOI of DENV2 in buffer. **(A)** P-selectin expression on platelets and **(B)** PAC-1 binding to platelet, **(C)** PS expression on platelets and **(D)** Microparticle generation by platelets treated with DENV were measured by flow cytometry. Data presented as mean ± SEM from 3 independent experiments. DENV increased the expression of P-selectin in a concentration-dependent manner (****P*<0.001). The DENV increased the PAC-1 binding in a concentration-dependent manner, ****P*<0.0001. The DENV increased PS expression in a concentration-dependent manner, ****P*<0.001. DENV increased the MP generation in a concentration-dependent manner, ***P<0.0001 (compared to DENV 0µl). **(D)** The higher copy number of viral genome was detected in platelet pellet when incubated with higher concentration of DENV2, ***p<0.0001.


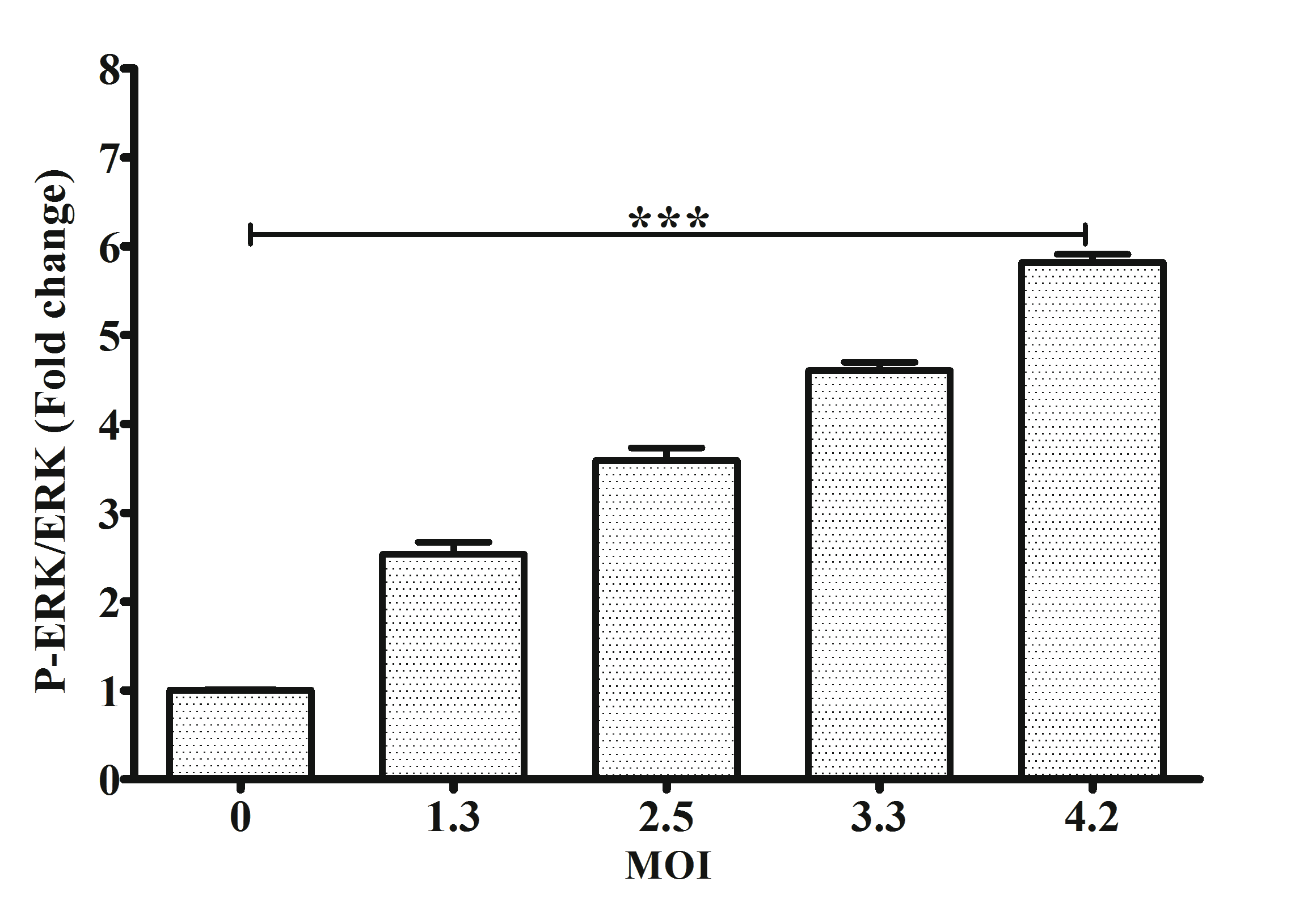

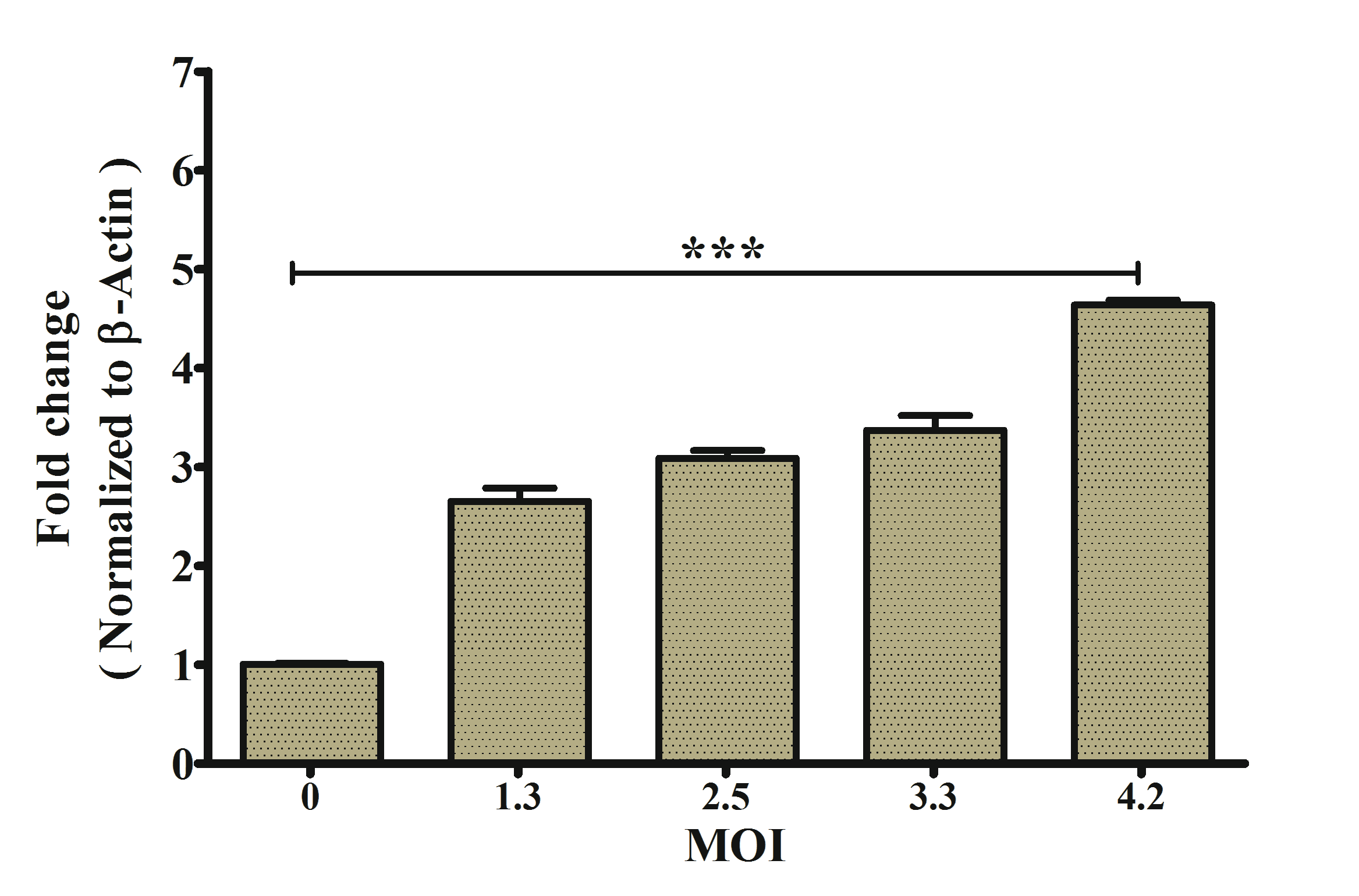

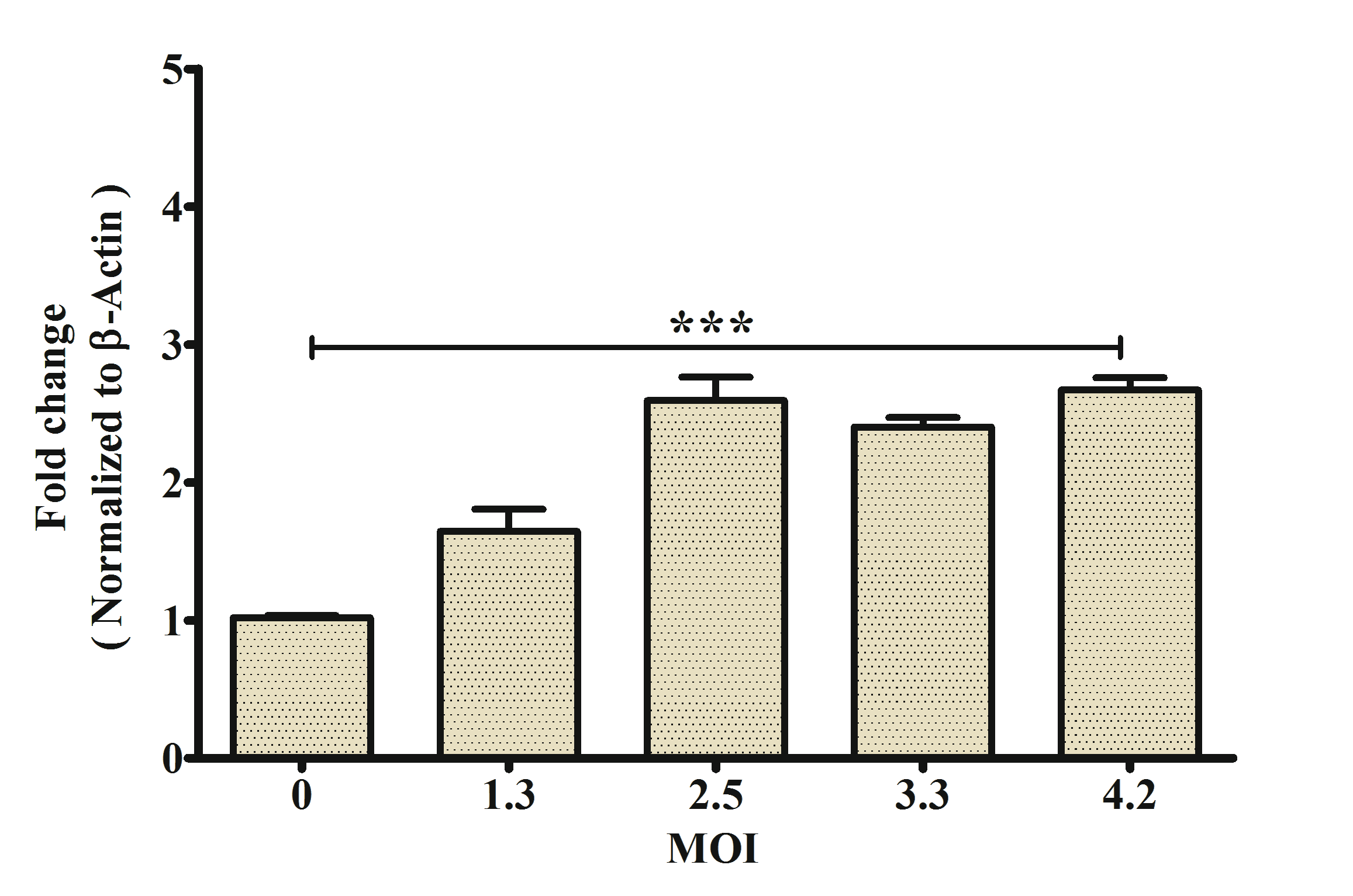


**A**

**B**

**C**


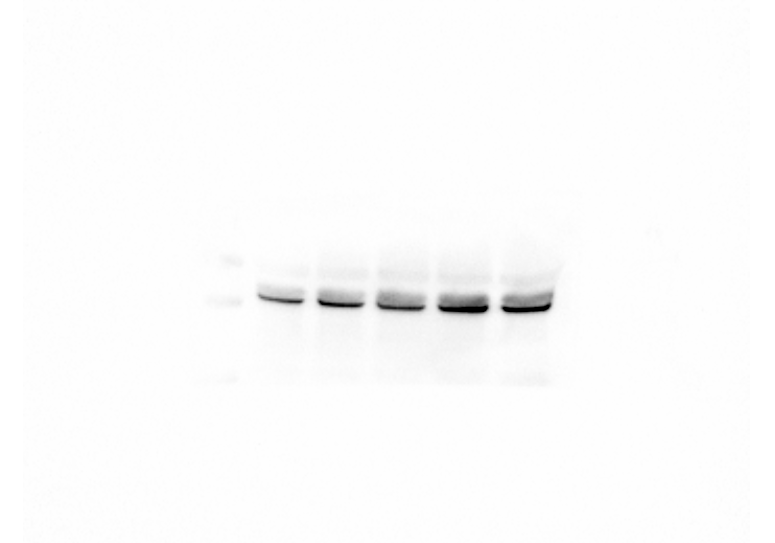


**MOI 0 1.3 2.5 3.3 4.2**

**44 kDA**

**P-ERK**


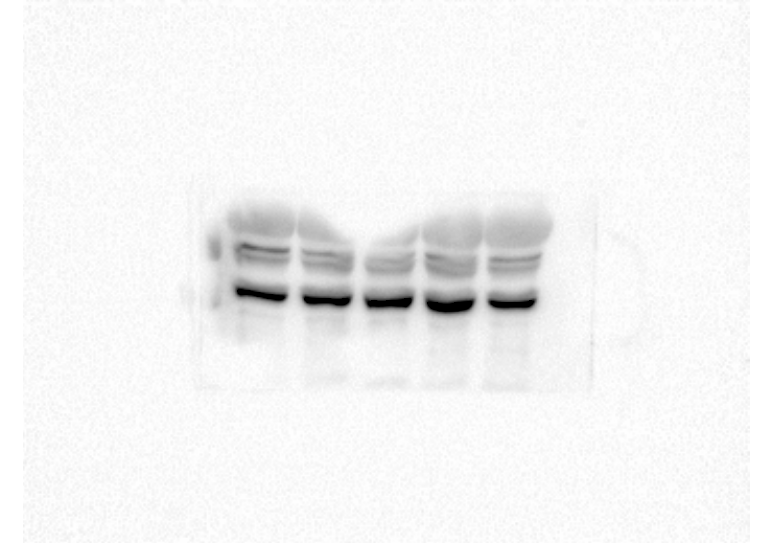


**MOI 0 1.3 2.5 3.3 4.2**

**ERK**

**44 kDA**


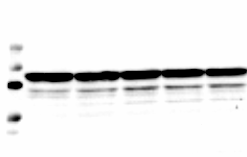


**MOI 0 1.3 2.5 3.3 4.2**

**Casp 9**

**Cleaved Casp 9**

**47 kDA**

**37 kDA**

**E**

**D**

**F**


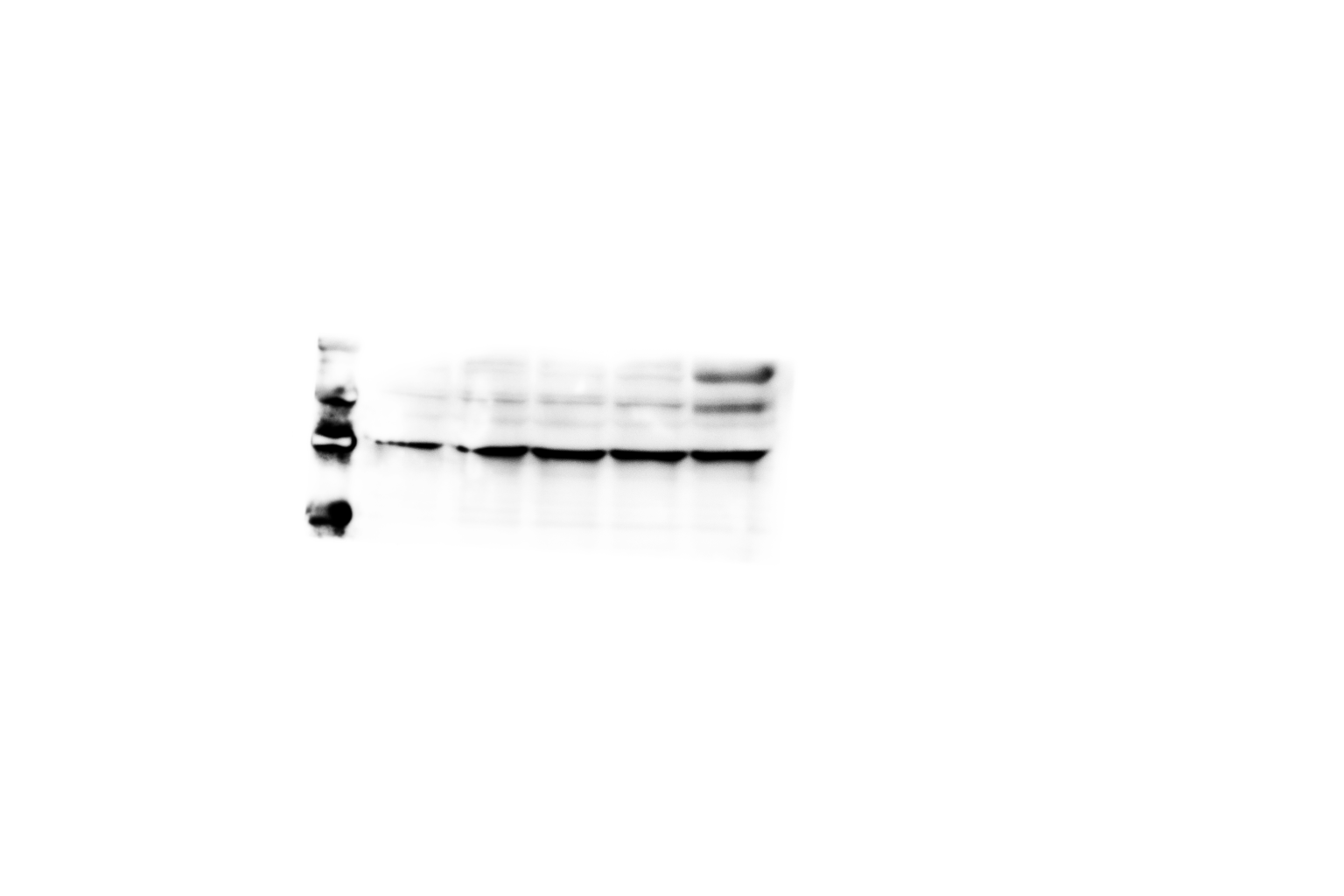


**MOI 0 1.3 2.5 3.3 4.2**

**Cyclophilin D**

**28 kDA**


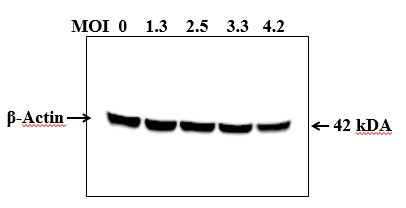


**G**

**H**

**Suppl. Fig. 4.** The densitometry of the immunoblots of **(A)** pERK/ERK (mentioned in Fig. 4C), ***p<0.0001 and **(B)** caspase 9, ***p<0.0001 and **(C)** cyclophilin D, ***p<0.0001 (mentioned in Fig. 4D) is measured as fold change from two independent experiments for each case. The uncut immunoblots showing the expression of ERK **(D)**, P-ERK **(E)**, Caspase 9 **(F)**, Cyclophilin D **(G)** and β-Actin **(H)**.


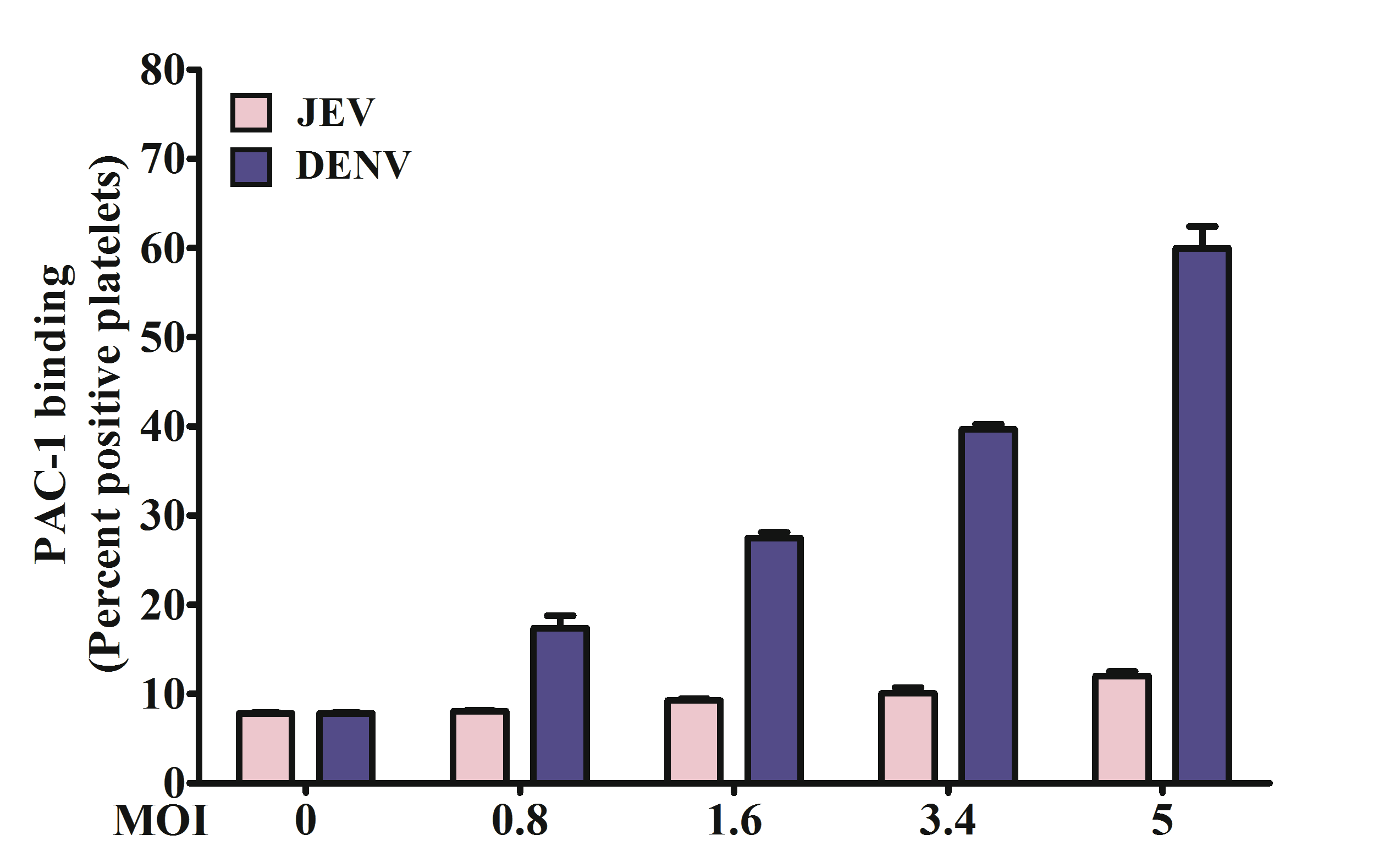

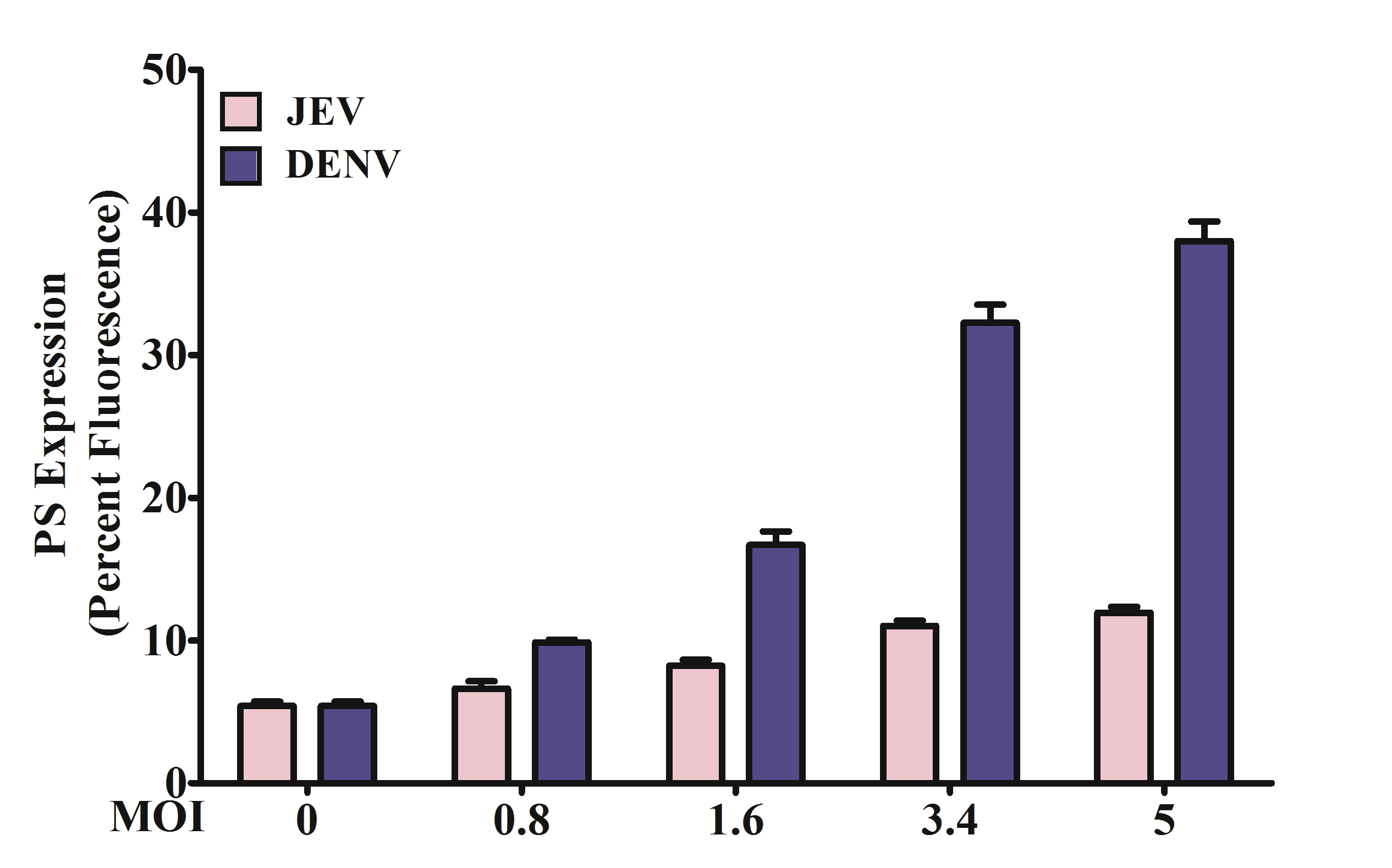

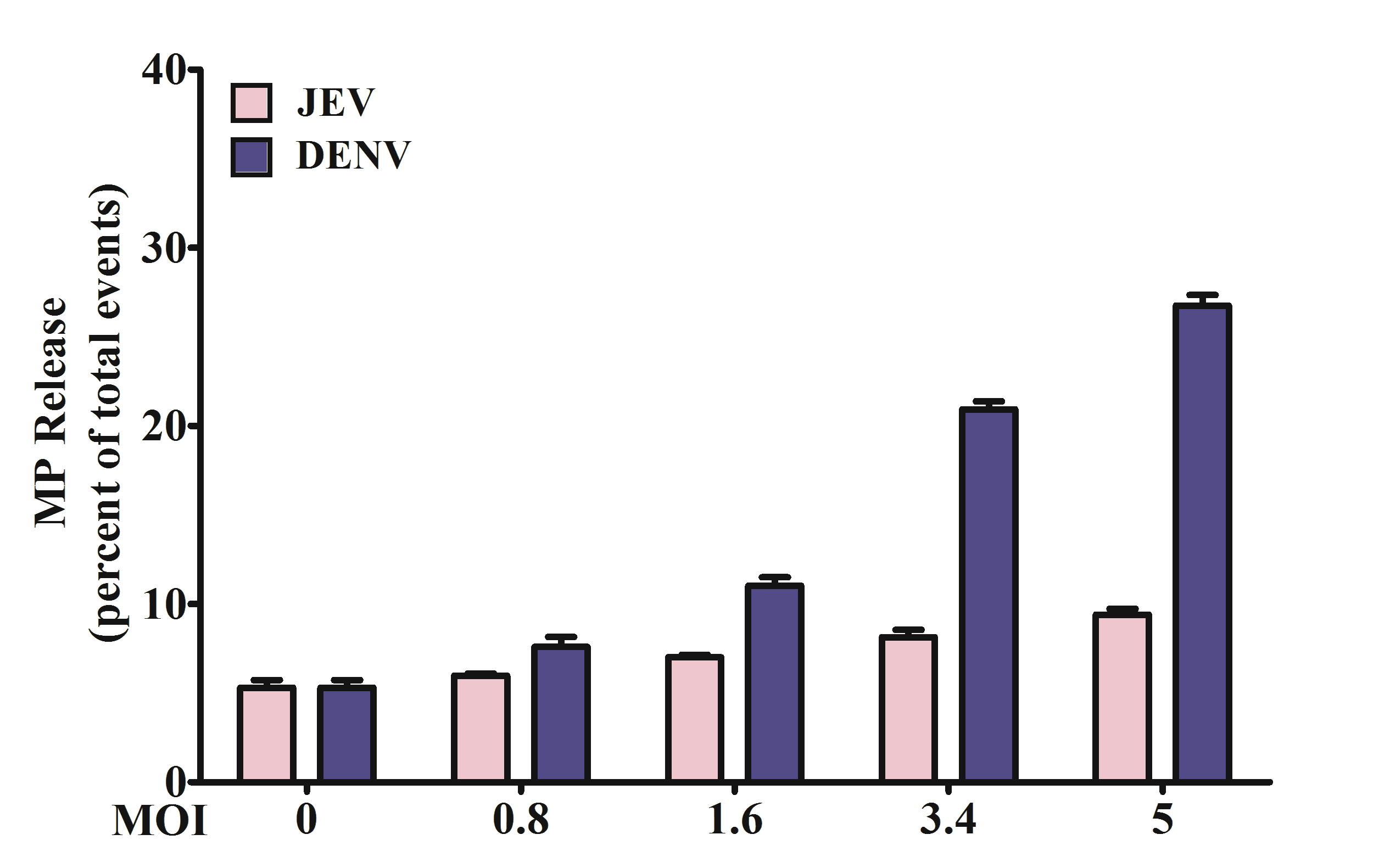


**A**

**B**

**C**

**Suppl. Fig 5. Platelet activation mediated by DENV2 & JEV *in vitro*.** Platelets rich plasma (PRP) were incubated with different MOI of JEV and DENV2. **(A)** PAC-1 binding to platelet, **(B)** PS expression on platelets and **(C)** Microparticle generation by platelets were measured by flow cytometry. Data presented as mean ± SEM from 3 independent experiments. The DENV increased the PAC-1 binding, PS expression and platelet-MP generation in a concentration-dependent manner, ****P*<0.0001, ****P*<0.001 and ***P<0.0001 respectively. JEV did not show significant effects.


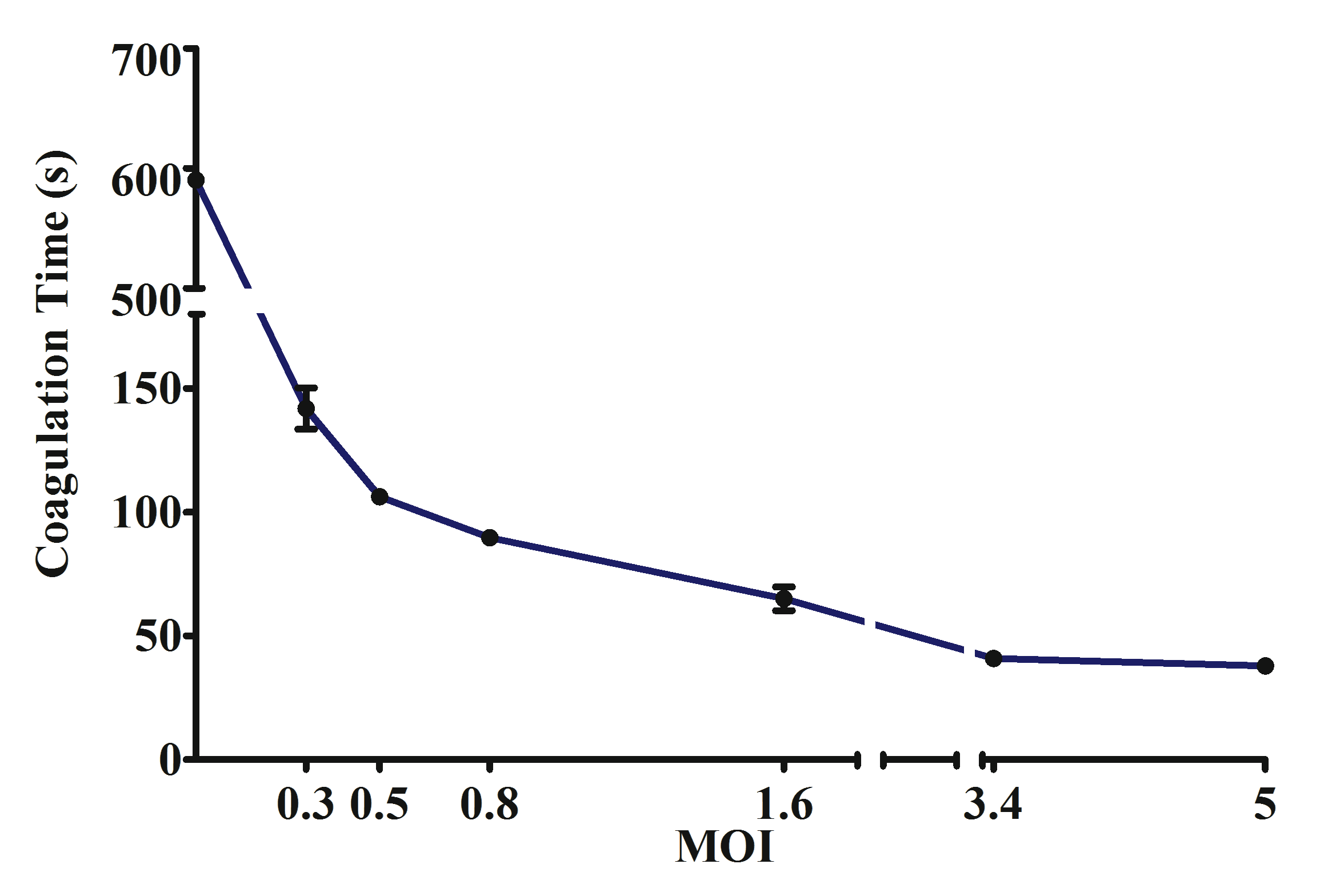


**Suppl. Fig. 6. Clot formation by platelets when treated with DENV2 *in vitro*.** Washed platelets were incubated with various MOI of DENV2. The clot formation time was measured in presence of plasma and coagulation reagents (Kaolin, 0.025M CaCl_2_). Data shows the mean ± SEM coagulation time from three independent experiments. The DENV2 treatment decreased the coagulation time in a concentration-dependent manner, p<0.0001 (One Way ANOVA).


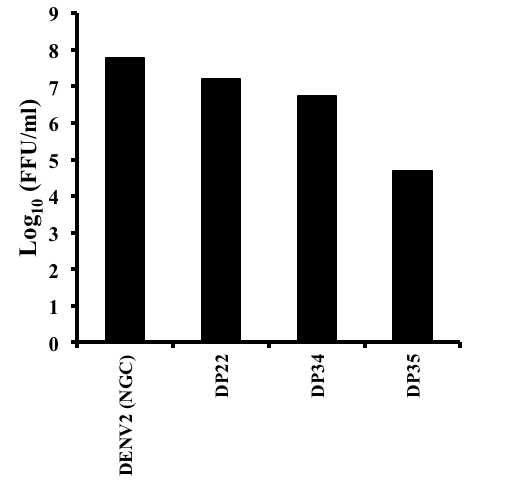


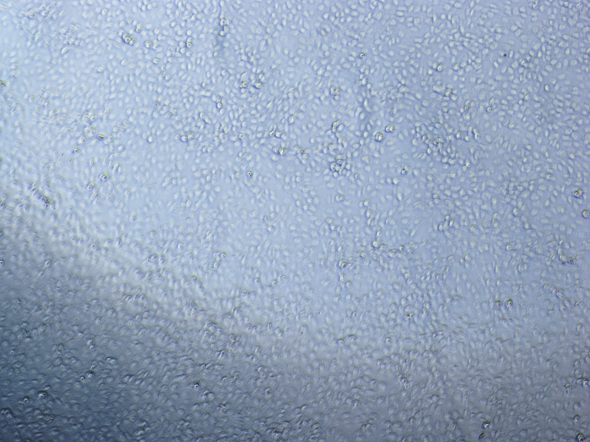


**Bright field**


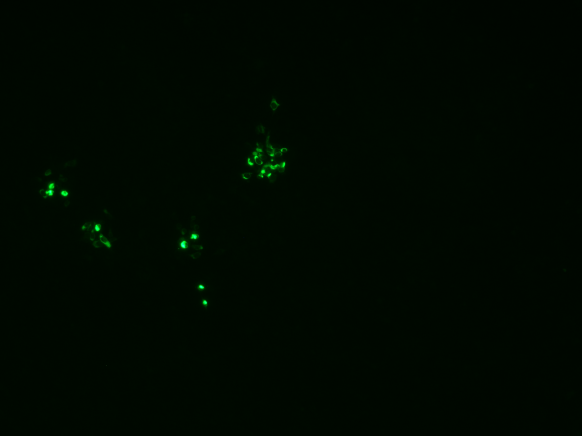


**Alexa-488**

**A**

**B**

**Suppl. Fig. 7: (A)** Plasma samples from DENV-infected patients (DP22, 34 and 35 at day 4) or DENV2 (NGC) grown *in* *vitro* in C6/36 cells were diluted in MEM-2% FCS and allowed to infect Vero cell monolayer for 2 hours; the inoculum was discarded and the infected cells maintained in MEM-10% FCS for 48 hours. Subsequently, the cells were fixed, immuno-stained with anti-DENV Mab8705 primary antibody followed by anti-Mouse Alexa-488 secondary antibody and observed under a fluorescent microscope. The numbers of fluorescent foci were counted and the infectious titer calculated as focus-forming unit per ml (FFU/ml) of inoculum. The logarithmic value to the base 10 of the FFU/ml for every inoculum was plotted. **(B)** A representative image of Vero cells in the FFU assay showing entire monolayer (bright field) or DENV-infected immuno-stained cells (Alexa-488).
